# Stakeholder perspectives towards diagnostic artificial intelligence: a co-produced qualitative evidence synthesis

**DOI:** 10.1016/j.eclinm.2024.102555

**Published:** 2024-03-22

**Authors:** Rachel Yi Ling Kuo, Alexander Freethy, Judi Smith, Rosie Hill, Joanna C, Derek Jerome, Eli Harriss, Gary S. Collins, Elizabeth Tutton, Dominic Furniss

**Affiliations:** a Nuffield Department of Orthopaedics, Rheumatology and Musculoskeletal Sciences, Oxford, UK; b Department of Plastic Surgery, Royal Devon and Exeter Hospital, Royal Devon University Healthcare NHS Foundation Trust, UK; c Bodleian Health Care Libraries, Oxford, UK; d Centre for Statistics in Medicine, Nuffield Department of Orthopaedics, Rheumatology and Musculoskeletal Sciences, Oxford, UK

**Keywords:** Artificial intelligence, AI, Qualitative, Systematic review, Qualitative evidence synthesis, Patient and public involvement

## Abstract

**Background:**

Diagnosis is a cornerstone of medical practice. Worldwide, there is increased demand for diagnostic services, exacerbating workforce shortages. Artificial intelligence (AI) technologies may improve diagnostic efficiency, accuracy, and access. Understanding stakeholder perspectives is key to informing implementation of complex interventions. We systematically reviewed the literature on stakeholder perspectives on diagnostic AI, including all English-language peer-reviewed primary qualitative or mixed-methods research.

**Methods:**

We searched PubMed, Ovid MEDLINE/Embase, Scopus, CINAHL and Web of Science (22/2/2023 and updated 8/2/2024). The Critical Appraisal Skills Programme Checklist informed critical appraisal. We used a ‘best-fit’ framework approach for analysis, using the Non-adoption, Abandonment, Scale-up, Spread, Sustainability (NASSS) framework. This study was pre-registered (PROSPERO CRD42022313782).

**Findings:**

We screened 16,577 articles and included 44. 689 participants were interviewed, and 402 participated in focus groups. Four stakeholder groups were described: patients, clinicians, researchers and healthcare leaders. We found an under-representation of patients, researchers and leaders across articles. We summarise the differences and relationships between each group in a conceptual model, hinging on the establishment of trust, engagement and collaboration. We present a modification of the NASSS framework, tailored to diagnostic AI.

**Interpretation:**

We provide guidance for future research and implementation of diagnostic AI, highlighting the importance of representing all stakeholder groups. We suggest that implementation strategies consider how any proposed software fits within the extended NASSS-AI framework, and how stakeholder priorities and concerns have been addressed.

**Funding:**

RK is supported by an NIHR Doctoral Research Fellowship grant (NIHR302562), which funded patient and public involvement activities, and access to Covidence.

**Research in context**

**Evidence before the study**

We searched PubMed and the Cochrane library from database inception to February 22, 2023 for papers published in English, using the search terms ‘qualitative evidence synthesis’ OR ‘qualitative review’ AND ‘artificial intelligence’ or ‘AI’ AND ‘healthcare’ OR ‘clinical’ OR ‘diagnosis’, to identify qualitative evidence syntheses on the topic of artificial intelligence in healthcare. We found a mixed-methods review, which described patient and public attitudes towards clinical artificial intelligence across three qualitative studies and six mixed-methods studies. They performed a thematic synthesis, developing six analytical themes: 1) AI concept, 2) AI acceptability, 3) AI relationship with humans, 4) AI development and implementation, 5) AI strengths and benefits and 6) AI weaknesses and risks.

**Added value of this study**

Our review builds on previous review findings by synthesising data on all stakeholder groups. We focus specifically on the application of AI to diagnostic tasks, and include 44 qualitative articles for analysis and interpretation. We have identified four distinct stakeholder groups: clinicians, patients and members of the public, researchers and leaders. Each stakeholder group holds a different perspective on diagnostic AI, with different factors influencing their decision to adopt, or not adopt the technology. Amongst stakeholder groups, patients and members of the public, researchers and leaders are under-represented in the primary literature. We tailor an established implementation science framework to diagnostic AI to inform future research, including two new subdomains of AI pertaining to the data used to train and test AI, and the setting of AI implementation.

**Implications of all the available evidence**

Implementation of diagnostic AI hinges on factors relating to the context of use, the technology, perceptions of added risks and benefits, the changes to the adopter system and organisation, and wider societal frameworks and context. Stakeholder groups have different priorities and key concerns regarding diagnostic AI. Future development and implementation strategies for diagnostic AI may consider how the software fits within the framework, and how to address stakeholder priorities to promote adoption. We suggest that future primary qualitative research focuses on the perspectives of under-represented stakeholder groups.

## Introduction

Diagnosis is a cornerstone of clinical decision-making. Worldwide, approximately 4.2 billion radiology studies are conducted each year.^1^ In the United States of America alone, 2–3 billion blood tests are performed annually.^2^ Rising demand for diagnostic services is coinciding with a global skills shortage: the World Health Organisation estimates a shortfall of 12.9 million healthcare workers by 2035, resulting in diagnostic delays and inaccuracies.^3^

Artificial intelligence (AI) holds promise to alleviate some of this burden, improving diagnostic efficiency, relieving increasing workforce pressures. AI has been reported to perform with high accuracy in diagnostic tasks across diverse pathologies, including fracture detection, cancer diagnosis, and retinal disease.^4^, ^5^, ^6^, ^7^ However, uptake remains limited.^8^

Understanding the factors influencing adoption of diagnostic AI hinges on complex interactions between the implementation environment, societal attitudes and the individual stakeholders involved in its development and eventual use. Insight into stakeholder perceptions of diagnostic AI, through qualitative research, is key to inform the acceptability, design, and delivery of AI tools across diverse settings.^9^

To address this, we performed a qualitative evidence synthesis (QES) of the literature exploring stakeholder perspectives of diagnostic AI. Our review is carried out as a collaboration between clinicians, researchers, and patient and public involvement (PPI) contributors. We synthesised our findings and developed a conceptual model describing stakeholder priorities and inter-relationships for sustained implementation of diagnostic AI.

## Methods

### Search strategy and inclusion criteria

The QES was prospectively registered (PROSPERO CRD42022313782) and reported using the Preferred Reporting Items for Systematic reviews and Meta-Analyses guideline (Supplementary Material 1).^10^ We identified relevant articles in a scoping search in PubMed, using these to develop search strategies for each database based on the combination of keywords relating to ‘artificial intelligence’ and qualitative research methods and methodology (Supplementary Material 2). We searched PubMed, Ovid MEDLINE, Ovid Embase, Scopus, CINAHL and Web of Science on 22/02/2023 and 8/02/2024 with no limitations on publication date. References were exported to EndNote 20 and duplicates were removed using the Systematic Review Accelerator Deduplicator tool.^11^

We included all peer-reviewed English language articles that investigated stakeholder perceptions of diagnostic AI and was primary qualitative or mixed-methods research.

We defined diagnostic AI as software designed to automate/semi-automate the diagnosis of any medical condition, based on health-data input of any modality: for example, radiological images. We did not place any restrictions on the output, or target-user of a software. We did not place restrictions on the level of autonomy exhibited by a diagnostic AI: i.e., a software designed to act as a diagnostic aid, compared to a software designed to replace a human clinician. We included all publications where participants discussed hypothetical or existing diagnostic AI, or where participants shared their opinions concerning AI within diagnostic medical specialties, such as radiology. We excluded publications where the software under discussion was rule-based (i.e., software that uses a set of immutable, predetermined rules).

Publications were excluded if participants did not explicitly discuss the use of diagnostic AI. For example, a study^12^ exploring midwives’ perceptions of AI was considered but ultimately excluded since participants did not discuss AI in the context of diagnosis.

### PPI collaboration

Our group consisted of academic clinicians (RK, DF), clinicians (AF), PPI contributors (RH, JS, DJ, JC, SD), qualitative researchers (ET), and statistician-methodologists (GC). We report our PPI experience using the Guidance for Reporting Involvement of Patients and the Public short-form checklist (GRIPP2-SF) (Supplementary Material 3).^13^

Five PPI contributors were recruited to diversify our groups’ perspectives. Meetings were held monthly, and written feedback gathered weekly. This facilitated a flattened hierarchy, in which members could challenge the preconceptions or bias of others. Reflections during the review process were recorded and collated after every meeting.

### Screening and data analysis

Identified articles were uploaded to Covidence^14^ web-based collaborative software. Titles and abstracts of each article were dual-screened for potential inclusion. Articles judged potentially eligible for inclusion by at least one author underwent full-text review.

A data extraction form was developed based on the COnsolidated criteria for Reporting Qualitative research (COREQ) checklist.^15^ The form was piloted and refined using three eligible publications employing different methods of data collection, before final review and approval.^16^, ^17^, ^18^

The Critical Appraisal Skills Programme (CASP) Qualitative Checklist was used for critical appraisal.^19^ Data extraction and critical appraisal was carried out independently for each included article. Discrepancies at any step were resolved through discussion.

We used a “best-fit” framework approach for qualitative analysis.^20^ This is a pragmatic, transparent approach to qualitative data synthesis, in which existing theoretical models are used for data analysis.^21^ We identified the Non-adoption, Abandonment, Scale-up, Spread, Sustainability (NASSS) framework as directly relevant to our review question.^22^ It aims to identify key challenges for adoption of healthcare technology across seven domains: the condition of interest, technology, value proposition, adopter system, organisation, wider context, and embedding/adaptation over time.

Qualitative data from each included publication were coded line-by-line and deductively mapped to NASSS framework domains and subdomains. We identified second-order constructs (researcher interpretations of data) as our primary data source. When studies included first-order constructs (direct quotations from study participants), we regarded these as chosen by the study researchers as illustrative of their interpretations. We therefore coded first-order constructs within domains/subdomains that were consistent with the associated second-order construct.

Microsoft Word and NVivo were used for data management.^23^ New codes were inductively developed for data not applicable to existing subdomains. Coding results were iteratively reviewed to develop consensus, and further refined after analysis of every five publications. The final framework and conceptual model were reviewed and approved by all authors.

### Ethical approval

Ethical approval was not required for this qualitative evidence synthesis.

### Role of the funding source

The funder of the study had no role in study design, data collection, data analysis, data interpretation, or writing of this report.

## Results

### Screening

**Fig. 1:**
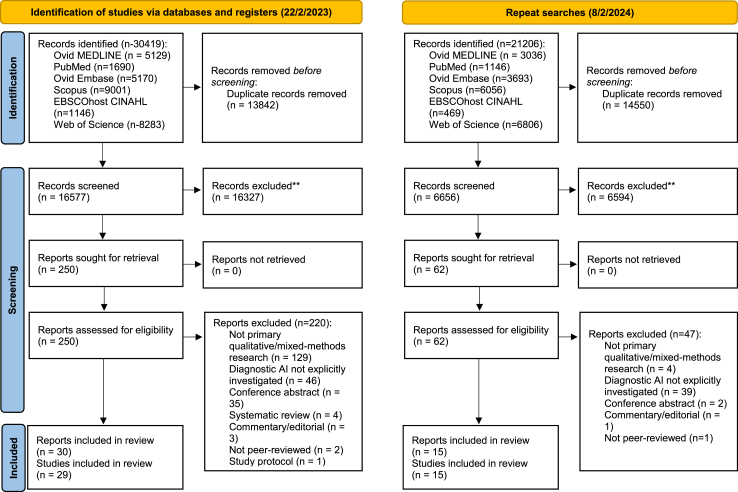
**PRISMA flow chart of study selection**.

Electronic searchers identified 30,419 potentially eligible articles. After deduplication 16,577 remained (Fig. 1). 16,327 were excluded after title and abstract screening. 250 articles underwent full-text screening (Supplementary Material 4). 29 studies met inclusion criteria. Two articles utilised the NASSS framework for data analysis.^24^^,^^25^ Findings of Richardson et al., were published in 2021 and 2022, involving the same participants but containing unique results.^26^^,^^27^ Both articles were collated to capture their findings and considered as one study, consistent with previous Cochrane QES’.^28^ We updated our searches on 8/02/2024, and identified a further 15 studies; in total, 44 studies were included (Fig. 1).

### Description of included studies

Table 1 summarises study characteristics. Across 44 studies, 689 participants were interviewed and 402 participated in focus groups. 31 studies conducted interviews,^16^^,^^24^^,^^25^^,^^31^^,^^32^^,^^34^^,^^36^, ^37^, ^38^^,^^40^, ^41^, ^42^, ^43^, ^44^, ^45^^,^^48^, ^49^, ^50^, ^51^^,^^53^^,^^55^^,^^57^^,^^59^, ^60^, ^61^, ^62^, ^63^, ^64^ nine conducted focus groups,^18^^,^^26^^,^^27^^,^^29^^,^^30^^,^^33^^,^^52^^,^^56^^,^^58^^,^^65^ five conducted ethnographic studies with interviews,^17^^,^^46^^,^^47^^,^^54^^,^^66^ and two conducted both interviews and focus groups.^35^^,^^47^ The mean number of participants in interview studies was 20 (IQR 15–24), and in focus groups 40(IQR 15–37).

**Table 1:** Characteristics of included studies.

Authors	Study design and methodology/data analysis	Method of data collection	Study population and sampling strategy	Number of participants	Participant characteristics	Topic of interest	AI studied	Main findings
Abuzaid et al. (2021)^29^	Mixed methods, thematic analysis	Focus groups	Magnetic Resonance Imaging (MRI) technologists, snowball sampling	98	NR	Radiology	Hypothetical use of AI in radiology	MRI technologists have an understanding of AI and believe it would improve MRI protocols, reduce scan time, enhance post-processing and play a role in image interpretation. They highlighted a need for bespoke for AI training and practical sessions.
Adams et al. (2020)^30^	Qualitative, thematic analysis	Focus groups	Patient and family advisors from urban and rural Canada, NR	17	11 (55%) female	Radiology	Hypothetical AI used in radiology	Patient perceptions were described in four themes: 1. Fear of the unknown, 2. Trust, 3. Human connection, 4. Cultural acceptability. Patient priorities for AI were described in five themes: 1. Improving access to imaging and reducing wait times, 2. Reducing time to diagnosis, 3. Increasing diagnostic accuracy, 4. Improving communication 5. Empowering patients.
Bergquist et al. (2023)^31^	Qualitative, NR	Semi-structured interviews	Mixed group of clinicians, researchers, and healthcare leaders in Sweden, purposive sampling	25	5 (20%) female; median age 53 years; 8 radiologists, 6 other medical professionals, 7 management, 4 engineers/developers	Radiology	Hypothetical AI used in radiology	Trustworthiness of AI is related to: 1. Reliability, 2. Transparency, 3. Quality verification and 4. Inter-organizational compatibility
Buck et al. (2022)^32^	Qualitative, grounded theory	Semi-structured interviews	General practitioners in Germany, convenience sampling	18	9 (50%) female, 9 (50%) male	General	Hypothetical AI used in medical diagnosis	Three determinants of GPs' attitudes towards AI: concerns, expectations, and minimum requirements of AI-enabled systems. Individual characteristics and environmental influences as the 2 conditional determinants of GPs' attitudes toward AI-enabled systems.
Carter et al. (2023)^33^	Mixed-methods, NR	Focus groups	Female members of the public aged 50–74 years old, Australia	50	Age: 20 (40%) aged 50–59, 21 (42%) aged 60–69, 9 (18%) aged 70–74 Personal history of breast cancer: 12 (24%) yes Education: 26 (52%) university educated	Radiology	Hypothetical AI used in radiology (breast cancer screening)	There is broad acceptance of the use of AI in breast screening, conditioned on human involvement and AI performance
Cartolovni, Malesevic and Poslon (2023)^34^	Qualitative, thematic analysis	Semi-structured interviews	Patients, clinicians, and healthcare leaders working in Croatia	38	Patients 11, clinicians 12, leaders 11; age range 18–65 years old	General	Hypothetical clinical AI	Four themes were developed: 1. The current state of healthcare and the patient-physician relationship, 2. Expectation of AI, 3. A synergistic effect between physicians and AI, 4. The future of healthcare and the patient-physician relationship
Chen et al. (2021)^35^	Qualitative, thematic analysis	Semi-structured interviews and focus groups	Radiologists and radiographers working in five NHS organisations in England, snowball sampling/convenience sampling	Interviews: 18 FG: 8	Interviews: 12 (60%) radiologists, 8 (40%) radiographers FG: 8 (100%) radiographers	Radiology	Hypothetical AI used in radiology	Considered responses to the use of AI in radiology in 1. Knowledge and 2. Attitudes, finding differences in attitudes towards AI between professional groups.
Drogt et al. (2022)^36^	Qualitative, NR	Semi-structured interviews	Mixed group of professionals working in the pathology department of two hospitals in the Netherlands, convenience sampling	24	15 (63%) pathologists, 17 (29%) laboratory technicians, 2 (8%) computer scientists	Pathology	Hypothetical AI used in pathology	Three recommendations for embedding AI in pathology: 1. Foster a pragmatic attitude toward AI development, 2. Provide task-sensitive information and training to health care professionals working at pathology departments, 3. Take time to reflect upon users' changing roles and responsibilities.
Faric et al. (2023)^37^	Qualitative, thematic analysis	Semi-structured interviews	Mixed group of clinicians, healthcare leaders, and patients across 5 hospitals in the UK, Belgium, and the Netherlands	39	12 patients: 10 (83%) female; 3 (25%) aged 40–50 years old, 3 (25%) aged 50–60 years old, 3 (25%) aged 60–70 years old, 3 (25%) aged 70–80 years old 25 clinicians/healthcare leaders: 6 (24%) female 2 healthcare leaders: 2 male	Radiology	AI in use for diagnosing lung nodules	Four main themes were developed: 1. Perceived drivers and benefits, 2. Design of the tool and integration, 3. Appropriation of the tool by expert labour, 4. Clinical governance, quality assurance, maintenance, and post-market surveillance
Fazakarley et al. (2023)^38^	Qualitative, thematic analysis	Semi-structured interviews	Clinicians and AI researchers in the UK	13	5 (38%) female; mean age 38 years old, SD 9.1 years; 9 (69%) White British; 2 (15%) mixed or multiple ethnicities; 1 (8%) Asian; 1 (8%) other 3 (23%) doctors, 4 (31%) nurses, 2 (15%) IT technicians, 4 (31%) AI developer/researcher	Cardiology	AI in use within a randomised-control trial, to diagnose coronary artery disease	Four themes were identified: 1. Positive perceptions of AI, 2. Potential barriers to using AI, 3. Concerns regarding AI use, 4. Steps needed to ensure the acceptability of future AI tools
Gillner (2024)^39^	Qualitative, thematic analysis	Semi-structured interviews, 1 focus group	A mixed group of AI providers across Europe and clinicians	Interviews: 17 FG: 5	Interviews: 17 AI researchers/leaders FG: 5 clinicians	General	Hypothetical clinical AI	Two major themes were developed: 1. Subsystems of complex healthcare systems, 2. Emergent practices of AI providers in healthcare
Haan et al. (2019)^40^	Qualitative, grounded theory	Semi-structured interviews	Patients attending the radiology department of a tertiary care, academic hospital in the Netherlands for CT chest and abdomen, purposive sampling	20	9 (45%) female; age 39–79 years old (mean age 64)	Radiology	Hypothetical AI used in radiology	Six key domains related to AI use in radiology: 1. Proof of technology, 2. Procedural knowledge, 3. Competence, 4., Efficiency, 5. Personal interaction, 6. Accountability
Hallowell et al. (2022)^41^	Qualitative, NR	Semi-structured interviews	Membership of the “Minerva Consortium” and personal contacts of the study authors, convenience/snowballing sampling	20	Expertise: 9 (45%) clinical genetics, 2 (10%) paediatric genetics, 5 (25%) bioinformatics, 2 (10%) commercial, 3 (15%) other	Rare disease	Hypothetical AI used in diagnosing facial dysmorphology	Discussion of the value of trust in using AI for dysmorphology, concluding that trust in AI is grounded in its reliability, competence and “intentions.”
Held et al. (2022)^42^	Qualitative, thematic content analysis	Semi-structured interviews	Mixed group of clinicians in Germany, convenience sampling	24	10 (42%) female; average year of birth 1971; 16 (67%) general practitioner, 3 (13) medical assistant, 5 (21%) ophthalmologists	Ophthalmology	Hypothetical AI used to diagnose diabetic retinopathy	Main determinants of implementation have been identified: personal attitude, organisation, time, financial factors, education, support, technical requirement, influence on profession and patient welfare.
Helenason et al. (2024)^43^	Mixed-methods, NR	Semi-structured Interviews	Primary care clinicians in Sweden	15	NR	Dermatology	AI used to diagnose skin lesions, proposed for use	Three major themes were identified: trust, usability and user experience and clinical context
Henry et al. (2022)^44^	Qualitative, grounded theory	Semi-structured Interviews	Mixed group of clinicians in a 285 bed acute-care, US hospital, purposive sampling	20	13 physicians (4 emergency department, 4 critical care, 5 general ward) and 7 nurses (3 emergency department, 4 critical care)	Sepsis	AI used to diagnose sepsis, in use by the institution	Themes identified included: lack of understanding of the difference between ML-based and conventional CDSS; ML-based systems play a supporting role; an overall willingness to trust AI despite lack of full understanding. Barriers highlighted included over-reliance on AI leading to deskilling.
Joshi et al. (2022)^45^	Qualitative, thematic content analysis	Semi-structured interviews	Hospital leaders in the USA	21	5 (24%) informatics leadership, 10 (48%) clinical leadership *e.g.*, CMO, 6 (29%) other executive leadership, convenience sampling	Sepsis	AI for diagnosis of sepsis, in use by the institution	Identified several barriers and facilitators to implementation of sepsis-detection AI, identifies consideration of workflow integration, and clinician buy-in as two key approaches to overcome identified barriers.
Jussupow et al. (2021)^46^	Qualitative, grounded theory	Semi-structured interviews and ethnography	Radiologists working in a hospital in Germany, with experience of using an AI system to diagnose stroke, snowball sampling	14	2 chief radiologists, 4 senior radiologists, 8 assistant radiologists; mean self-reported diagnostic confidence (1–10) ranging from 4.3–10.0	Radiology	AI for stroke diagnosis, in use at the institution	Described three patterns of AI use. “Sensedemanding”radiologists will evaluate AI results in both confirming and disconfirming AI, “Sensegiving” radiologists will reinforce use if AI confirms their findings. “Sensebreaking” radiologists find no benefit from AI.
Kim et al. (2024)^47^	Qualitative, ethnography with abductive reasoning	Semi-structured interviews and ethnography	Mixed group of clinicians and healthcare leaders working at a hospital in the Netherlands	Ethnographic observation over 3 years; 18 interviews	NR	Radiology	15 individual AI pipelines in use, for cross-specialty diagnostic tasks	Three key themes were developed to inform AI implementation: 1. Technology level, 2. Workflow level, 3. People and organisational level
King et al. (2023)^48^	Qualitative, framework approach	Semi-structured interviews	Pathologists employed in UK hospitals, purposive sampling	25	20 pathology consultants/attendings, 5 pathology trainees. 14 (70%) male, 11 (30%) female.	Pathology	Hypothetical AI used in pathology	Required features of AI identified by pathologists were trustworthiness and explainability, usability and workflow integration. Key contextual information and concerns about AI included the context of AI deployment, pathologists involvement with AI development, liability, evaluation and validation of AI and resources for AI.
Lebovitz et al. (2022)^17^	Qualitative, grounded theory	Semi-structured interviews and ethnography	Radiologists working in three departments utilising diagnostic AI, NR	33 + 500 h of ethnographic observation	NR	Radiology	AI in use for diagnosing breast cancer, classifying lung nodules and determining bone age	Only radiologists diagnosing lung cancer engaged with AI tools, despite high accuracy of all AI tools in the study. Explainability of AI is a necessary feature for clinician engagement, but on its own is permissive rather than sufficient.
Lombi and Rossero (2023)^49^	Qualitative, template analysis	Semi-structured interviews	Radiologists working in a mixture of private and public hospitals in Italy, purposive sampling	12	1 (8%) female, age range 36–64 years, 5 (42%) employed by private hospitals	Radiology	Hypothetical AI used in radiology	Three themes were developd: 1. ‘It will take time’ 2. ‘This is what being a radiologist means’ 3. ‘Don't be a DIY diagnostician!’
Massey et al. (2023)^50^	Mixed-methods, content analysis	Semi-structured interviews	Otolaryngologists, working in the USA, purposive sampling	19	11 (58%) general otolaryngologists, 8 (42%) subspecialty rhinologists; 11 (58%) practicing in an academic setting.	Radiology	Hypothetical AI used in radiology for sinus CT interpretation	Six themes were identified: 1. Conventional reporting was indispensable for extra-sinus analysis, 2. Relationship with radiologist dictates trust in reporting, 3. Clinicians were open to utilizing AI, 4. Standardization of reporting was valued, 5. Anatomical analysis was preferred over descriptive assessments, 6. Trust in AI could be improved with additional validation in the literature
Mosch et al. (2022)^51^	Qualitative, thematic analysis	Semi-structured interviews	Mixed group of participants with expertise in the field of AI in medicine, medical education, and training, purposive sampling	24	Professional background: 15 (63%) medical, nine (38%) computer science, 3 (13%) medical education, 8 (23%) other	General	Hypothetical clinical AI	Three themes were developed: 1. Specific tasks of physicians will be taken over by AI systems, 2. AI will not replace physicians, 3. Ways of work: AI will transform how healthcare is delivered.
Nelson et al. (2020)^16^	Qualitative, grounded theory	Semi-structured interviews	Patients attending general dermatology clinics and melanoma clinics at a hospital in the USA, purposive sampling	48	26 (54%) female; mean (SD) age 53.3 (21.7) years old; 16 (33%) history of melanoma, 16 (33%) history of non-melanomatous skin cancer, 16 (33%) no history of skin cancer; 45 (94%) White, 2 (4%) American Indian or Alaskan Native, 1 (2%) African American	Dermatology	Hypothetical AI used in dermatology for skin lesion classification	Patients describe a preference for AI as an assistive tool, rather than a replacement for a clinician. Increased diagnostic speed, accuracy and healthcare access were commonly perceived benefits of AI, but perceived risks included increased patient anxiety, AI errors and loss of human interaction.
Ng et al. (2022)^52^	Qualitative, Phenomenology/thematic analysis	Focus groups	Radiographers working in public institutions in Singapore, purposive sampling	22	11 (50%) female; age 23–42 years old (median 30.5 years); working experience 1–18 years (median six years)	Radiology	Hypothetical AI used in radiography	Four themes were developed from the data: 1. Knowledge of AI and its applications, 2. Perceptions on the use of AI in radiographic practice, 3. Patients' perceptions as viewed by radiographers, 4. Prospective applications and expectations of AI.
Pelayo et al. (2023)^53^	Qualitative, framework analysis	Semi-structured interviews	Latinx patients with T2DM at a single health center in the USA	20	12 (60%) female; mean age 59.8, range 14	Ophthalmology	Hypothetical AI used to diagnose diabetic retinal disease	Patients strongly prefer human review rather than AI; if AI is integrated it should be as a tool rather than a replacement
Prakash et al. (2021)^54^	Qualitative, thematic analysis	Netnography, semi-structured interviews	Radiologists working in India, purposive sampling	15	5 (33%) female; mean age 40.7 years old, range 28–62	Radiology	Hypothetical AI used in radiology	Themes were developed from qualitative data: 1. Perceived threat, 2. Medico-legal risk, 3. Performance risk, 4. Performance expectancy, 5. Trust, 6. User resistance
Pumplun et al. (2021)^24^	Qualitative, directed content analysis	Semi-structured interviews	Mixed group of AI experts, with detailed knowledge of clinical processes and AI, theoretical sampling approach	22	5 (23%) clinicians, 8 (36%) clinicians with leadership roles, 9 (25%) managers or IT staff; between 3 and 40 years of work experience	General	Hypothetical AI used in diagnosis	Developed a maturity model to score the readiness of a clinic for AI adoption, spanning three dimensions: organisation, adopter system and patient data.
Rabinovich et al. (2022)^55^	Mixed-methods, NR	Structured interviews	Mixed group of clinicians in a hospital in Argentina, with experience of using diagnostic AI, NR	6	3 (50%) emergency physicians and 3 (50%) radiology residents	Radiology	AI in use in the institution, for diagnosing pneumothoraces, rib fractures, pleural effusions, and lung opacities on chest radiographs	Participants in general had positive experiences with using the diagnostic AI. They describe using it as a second opinion, to reduce human error, and valued its use in diagnostic confirmation.
Redrup Hill et al. (2023)^56^	Qualitative, NR	Focus groups	Mixed group of patients/clinicians, researchers and healthcare leaders	31	4 software developers, 7 pathologists, 11 leaders, 9 patients/clinicians	Pathology	Existing AI to diagnose Barett's oesophagus or adenocarcinoma from pathology specimens	Six themes were developed: 1. Risks and potential harms, 2. Impacts on human experts, 3. Equity and bias, 4. Transparency and oversight, 5. Patient information and choice, 6. Accountability, moral responsibility and liability for error
Richardson et al. (2021)^26^	Qualitative, grounded theory	Focus groups	Patients who had a recent primary care visit at a large academic health centre in the USA, convenience sampling	87	49% female; average age 53.5 years old; 93% white and 94% non-Hispanic/Latino; 87% education level higher than a high school degree; 20% employment history in technology or computer science; 45% employment history in healthcare/health science	General	Hypothetical clinical AI, using case studies to ground discussion	Description of six themes: excitement about healthcare AI but needing safety assurances, and expectation for clinicians to ensure AI safety, preservation of patient choice and autonomy, concerns about healthcare costs and insurance coverage, ensuring data integrity, and the risks of technology-dependent systems.
Richardson et al. (2022)^27^	As above	As above	As above	As above	As above	As above	As above	Developed a conceptual framework for understanding how patients evaluate healthcare AI, based on patient experiences (with illness, health technology, relationship with clinicians, social context and familiarity with technology), beliefs (about healthcare and technology) and attitudes towards AI in healthcare (attitude formation, perceived acceptability and support for development).
Robertson et al. (2023)^57^	Mixed-methods, NR	Semi-structured interviews	Patients recruited from cardiac clinics in Tucson, Arizona; convenience sampling	24	16 (67%) female; age range 19–92 years old; 10 (42%) White, 8 (33%) Hispanic, 3 (13%) Black, 2 (8%) Native American, 1 (4%) Asian, 7 (29%) University education	General	Hypothetical clinical AI	Narrative overview of qualitative data; patients discussed fallibility of AI systems, trust related to healthcare systems, knowledge of AI in use, confidence in human physicians and religious belief
Sangers et al. (2021)^58^	Qualitative, thematic content analysis	Focus groups	Members of the public who took part in a customer panel of a Dutch health insurer, and social media platforms; purposive sampling	27	18 (67%) female; mean age 37.3 years, range 19–73; all use a smartphone at least every half day; 20 (74%) no history of skin cancer, 4 (15%) personal history of skin cancer, 3 (11%) family history of skin cancer	Dermatology	Hypothetical AI used in diagnosing skin cancer	Barriers to using AI apps for skin cancer diagnosis were: perceived lack of value, perception of untrustworthiness, preference for humans, concerns about privacy, complex user interface and increased costs. The facilitators were high perceived value, transparent and trustworthy identity of AI developers, endorsement by clinicians and regulatory bodies, easy to use interface and low costs.
Sangers et al. (2023)^18^	Qualitative, grounded theory	Focus groups	Dutch dermatologists and GPs identified through social media and via specialty newsletters, purposive sampling	33	Mean age 35.6 years, range 31–62; 17 (52%) female; 17 (52%) general practitioner, 16 (49%) dermatologist	Dermatology	Hypothetical AI used in diagnosing skin cancer	Dermatologists and GPs described preconditions for implementation: adequacy of algorithms, sufficient usability and accessibility, validation and regulation/clear liability, national guidance; they described benefits including improved health outcomes, care pathways and education. They described perceived barriers as doubts about AI accuracy, exacerbation of health inequalities, fear of replacement by AI, extra time taken to use AI and commercialization and privacy concerns.
Satterfield et al. (2019)^59^	Qualitative, thematic analysis	Semi-structured interviews	3 groups of researchers: diagnosis, AI, “Learning Health Systems”, NR	32	18 (56%) from the “improving diagnosis” research group, 6 (19%) from AI research, 8 (25%) from the “Learning Health Systems” group	General	Hypothetical AI used in diagnosis	There is limited collaboration between the research communities, and the authors emphasise the importance of forming a multi-disciplinary “learning community” to ensure uptake of AI in diagnosis.
Scheetz et al. (2021)^60^	Mixed-methods, thematic analysis	Semi-structured interviews	Mixed group of clinicians, including doctors and AHP, with experience of using an AI tool to screen for diabetic retinopathy, in outpatient clinicians in Australia, convenience sampling	8	3 (37.5%) male doctors, 5 (62.5%) female AHP	Ophthalmology	AI to screen for diabetic retinopathy	Participants agreed that the AI tool was easy to use and interpret, but reported challenges in explaining findings to patients, and allocating enough time to use the tool. They reported the requirement for validation of any AI tool to increase trust, and the value of AI was felt to be reducing the burden on individual clinicians.
Sibbald et al. (2022)^61^	Qualitative, content analysis	Semi-structured interviews	Emergency department physicians with personal experience of using an AI tool to support differential diagnosis (EDS) at triage, purposive sampling	13	2 (15%) female; 5 (38%) <5 years of practice, 4 (31%) 5–10 years, 1 (7%) 11–20 years, 3 (23%) >20 years; 6 (46%) family medicine specialists with subspecialisation in emergency medicine, 7 (54%) emergency medicine specialists	Emergency medicine	AI in use to generate differential diagnosis for emergency medicine triage	Four themes were identified: 1. The quality of EDS was inferred from the scope and prioritization of the diagnoses, 2. Trusting EDS differential diagnoses was linked to varied beliefs around the diagnostic process and potential for bias, 3. Who benefits? Not me, 4. Information flow between EDS and the Electronic Medical Record.
Strohm et al. (2020)^25^	Qualitative, NR	Semi-structured interviews	Mixed group of radiologists, managers, implementation consultants and data scientists with experience using an AI for automating bone maturity assessments (BoneXpert), sampling for maximal variation	24	20 (83%) radiologists, 5 of which have a dual role as data scientists/managerial, 4 (17%) managers	Radiology	Hypothetical AI used in radiology, with reference to BoneXpert, an AI developed by a commercial company (Visiana) that automated bone maturity assessments using paediatric hand X-rays	Using the NASSS framework, identified facilitating and hindering factors for AI implementation, with one of the most important barriers identified as the non-acceptance of AI by clinicians.
Townsend et al. (2023)^62^	Mixed-methods, thematic analysis	Semi-structured interviews	Clinicians with current or previous emergency department roles, located in the UK	9	4 (44%) female; age range 20–59 years; experience in emergency medicine range 1 month–22 years	Emergency department	AI in use to generate differential diagnosis for emergency medicine triage	The overarching theme is ‘trust’, with five subthemes: 1. Social, 2. Legal, 3. Ethical, 4. Empathetic, 5. Cultural
van Cauwenberge et al. (2022)^63^	Mixed-methods, thematic analysis	Think-aloud interviews	Physicians working in a large tertiary care academic hospital in the Netherlands, purposive sampling	30	16 (53%) female; 7 (23%) in training, 8 junior (27%), 15 (50%) senior	General	Hypothetical AI for general clinical and diagnostic support	Four themes were developed: 1. Transparency, 2. Obstructivity, 3. Type of problem, 4., Certainty of advice
Wenderott et al. (2024)^64^	Qualitative,	Semi-structured interviews	Radiologists and radiology residents in a hospital in Germany, convenience sampling	12	8 (67%) 2–4 years of work experience; 5 (42%) worked in department <1 year, 4 (33%) worked in department 1–3 years	Radiology	AI in use to diagnose prostate lesions on MRI	Findings were categorised into AI benefits/risks, barriers/facilitators, external factors influencing AI adoption and contradictory statements
Winter and Carusi (2022)^65^	Qualitative, thematic analysis	Focus groups	Mixed group of professionals involved developing AI for clinical use, and patients/carers with lived experience of pulmonary hypertension, NR	21, split into two FG (10, 11)	FG1: 4 (19%) computer scientists, 4 (19%) clinicians,2 (10%) researchers, 1 (4%) patient representative FG2: 6 (29%) patients, 4 (19%) carers, 1 (4%) patient representative	Respiratory	Hypothetical AI used to diagnose pulmonary hypertension	Four themes were developed: 1. AI can result in early diagnosis, 2. Early diagnosis outweighs data risks of privacy and reuse, 3. Responsibility lies with specialist clinicians, 4. AI will result in deskilling of professionals.
Winter and Carusi (2022)^66^	Qualitative, thematic analysis	Semi-structured interviews and ethnography	Mixed group of professionals involved in the development of a screening algorithm for pulmonary hypertension, NR	3	2 (67%) researchers, 1 (33%) clinician	Respiratory	AI under development to screen for pulmonary hypertension.	Collaboration between clinicians and researchers is encouraged, particularly in 1. Querying datasets, 2. Building the software and 3. Training the model.

NR, Not reported.

Four distinct stakeholder groups were described: patients/the public, clinicians, researchers, and healthcare leaders. Twenty studies focussed on clinician perspectives,^17^^,^^18^^,^^29^^,^^32^^,^^35^^,^^42^^,^^44^^,^^46^^,^^48^^,^^50^^,^^52^^,^^54^^,^^55^^,^^60^^,^^61^^,^^63^ eight on patients,^16^^,^^26^^,^^27^^,^^30^^,^^33^^,^^40^^,^^53^^,^^57^^,^^58^ and one on researchers^59^ and healthcare leaders/experts.^45^ Fourteen focussed on mixed groups.^24^^,^^25^^,^^31^^,^^34^^,^^36^^,^^38^^,^^39^^,^^41^^,^^47^^,^^51^^,^^56^^,^^65^, ^66^, ^67^ Of 29 studies exploring clinician perspectives, 26 included doctors,^17^^,^^18^^,^^24^^,^^25^^,^^31^^,^^32^^,^^34^, ^35^, ^36^, ^37^, ^38^, ^39^^,^^41^^,^^42^^,^^44^^,^^46^, ^47^, ^48^^,^^55^^,^^56^^,^^59^, ^60^, ^61^^,^^63^^,^^65^^,^^66^ six included allied healthcare professionals,^29^^,^^35^^,^^37^^,^^42^^,^^52^^,^^60^ and two included nurses.^38^^,^^44^

Twenty-six studies were conducted in Western Europe (Belgium, Germany, The Netherlands, Sweden, Switzerland, Italy, and the United Kingdom),^18^^,^^24^^,^^25^^,^^31^^,^^32^^,^^35^, ^36^, ^37^, ^38^, ^39^, ^40^, ^41^, ^42^, ^43^^,^^46^, ^47^, ^48^, ^49^^,^^51^^,^^56^^,^^58^^,^^63^, ^64^, ^65^, ^66^ eleven in North America (Canada and the United States of America),^16^^,^^17^^,^^26^^,^^27^^,^^30^^,^^44^^,^^45^^,^^50^^,^^53^^,^^57^^,^^59^^,^^61^ two in Australia,^60^ and one in each of Argentina^55^^,^^60^ Croatia,^34^ India,^54^ Singapore,^52^ and the United Arab Emirates.^29^

Sixteen studies focussed on diagnostic AI applied to radiology,^17^^,^^25^^,^^29^, ^30^, ^31^^,^^33^^,^^35^^,^^40^^,^^46^^,^^47^^,^^49^^,^^52^^,^^54^^,^^55^^,^^64^^,^^67^ three in each of on dermatology,^16^^,^^18^^,^^58^ ophthalmology,^42^^,^^53^^,^^60^ pathology,^36^^,^^48^^,^^56^ two in each of sepsis detection,^44^^,^^45^ and respiratory disease,^65^^,^^66^ emergency medicine^61^^,^^62^ and one in each of cardiology,^38^ otolaryngology,^50^ and rare disease.^41^ Ten studies investigated the application of diagnostic AI in general.^24^^,^^26^^,^^27^^,^^32^^,^^34^^,^^39^^,^^43^^,^^51^^,^^57^^,^^59^^,^^63^

For data analysis, sixteen studies employed thematic analysis,^29^^,^^30^^,^^34^^,^^35^^,^^37^, ^38^, ^39^^,^^49^^,^^51^^,^^54^^,^^59^^,^^60^^,^^62^^,^^63^^,^^65^^,^^66^ seven took a grounded theory or modified grounded theory approach,^16^^,^^17^^,^^26^^,^^27^^,^^32^^,^^40^^,^^44^^,^^46^ seven performed content analysis,^18^^,^^42^^,^^45^^,^^50^^,^^58^^,^^61^^,^^64^ four used framework analysis,^18^^,^^24^^,^^25^^,^^42^^,^^45^^,^^48^^,^^53^^,^^58^^,^^61^ one performed template analysis,^49^ and one took a phenomenological approach with thematic analysis.^52^ Nine do not explicitly record their methodological approach but describe an inductive process for data analysis.^31^^,^^33^^,^^36^^,^^41^^,^^43^^,^^47^^,^^55^, ^56^, ^57^

Adherence with the COREQ reporting checklist was variable, as was quality appraisal (Supplementary Material 5). In general, reflexivity statements were incomplete. Only eight studies included reflections on interviewer characteristics, considering how this might influence data analysis.^16^^,^^37^^,^^38^^,^^44^^,^^48^^,^^52^^,^^61^^,^^64^ Reporting of study design and data analysis were more consistent, but there was heterogeneity in description of participant demographics. 33 studies reported participant occupation,^17^^,^^18^^,^^24^^,^^25^^,^^31^^,^^32^^,^^34^, ^35^, ^36^, ^37^, ^38^, ^39^^,^^41^^,^^42^^,^^44^, ^45^, ^46^^,^^48^, ^49^, ^50^, ^51^, ^52^^,^^54^, ^55^, ^56^^,^^59^, ^60^, ^61^, ^62^, ^63^, ^64^, ^65^, ^66^ but only 23 reported sex,^16^^,^^18^^,^^26^^,^^27^^,^^30^, ^31^, ^32^, ^33^, ^34^^,^^37^^,^^38^^,^^40^^,^^42^^,^^48^^,^^49^^,^^52^, ^53^, ^54^^,^^57^^,^^58^^,^^60^, ^61^, ^62^, ^63^ and eighteen age.^16^^,^^18^^,^^26^^,^^27^^,^^31^, ^32^, ^33^, ^34^^,^^37^^,^^38^^,^^40^^,^^49^^,^^52^, ^53^, ^54^^,^^57^^,^^58^^,^^62^

### ‘Best-fit’ framework

Across 44 studies, 2540 items were extracted, and allocated to domains/subdomains of the NASSS framework. We found that one domain and two subdomains (7: embedding and adaptation over time, 2D: technology supply model, 4C: carers) were not represented in the data. We adapted the framework to include two new subdomains (1C: Nature of setting, 2D: Nature of data used to train AI), 43 themes, and 33 subthemes specific to adoption of diagnostic AI.

We present an extended NASSS-AI framework, consisting of six domains, 20 subdomains, and 43 themes (Table 2), providing in-depth analysis of frequently discussed elements of the framework.

**Table 2:** Extended NASSS-AI framework.

Domain	Subdomain	Themes	Subthemes	Illustrative quotation
1. Condition	A. Nature of condition or task	i. Administrative tasks		“*[Administrative tasks] are trivial. They are very easy and should, obviously, be integrated [in the system]*.” (Doctor, van Cauwenberge et al., 2022^63^)
		ii. Clinical or clinical support tasks		“*Clinical decision support … would really help us in terms of ensuring that all the requests are appropriate.”* (Radiographer, Ng et al., 2022^52^)
	B. Co-morbidities or socio-cultural differences	i. Clinical context		“*Diagnosis is totally context-dependent and I don't think you can teach a machine how to interpret clinical context, it's too nebulous a concept actually … context is everything.”* (Doctor, King et al., 2023^48^)
		ii. Multi-modal diagnosis		“*A problem in pathology diagnostics is the complexity … you also need to integrate clinical data to reach a diagnosis.*” (Doctor, Drogt et al., 2022^36^)
	C. Nature of setting	i. End-user		“*Generalists diagnose a wide range of pathologies and apply a diverse set of grading rules; interviewees felt tools that supported this would enable generalists to deal with a greater range of work to a higher degree of accuracy.”* (King et al., 2023^48^)
		ii. Healthcare setting		*“[AI] should be made available mainly for practices specialising in diabetes, preferably in rural areas, for reasons of cost-effectiveness.”* (Held et al., 2022^42^)
2. Technology	A. Material features	i. AI lacks humanity		*“Those people who are broken by the idea of getting something scary like cancer, where do they turn?”* (Patient, Nelson et al., 2020^16^)
	B. Type of data generated	i. Quality of diagnosis	a. Diagnostic accuracy	“*Accuracy is the absolute most important thing and that they should never think about bringing in something to save money or create convenience. That the accuracy is better, at least as good, but definitely better than what's there, because that's the most important thing.”* (Carter et al., 2023^33^)
			b. Diagnostic credibility	*“A data scientist, reflected that clinicians might come to regard [AI tools] as useful, if they produce results that agree with them, but may ignore them or come to distrust them, if the results they produce are in disagreement with their assessment.”* (Hallowell et al., 2022^41^)
		ii. Explainability and transparency	a. Level of explainability	*“What is it telling me to look at? At this tissue? It looks just like the tissue over here, which is perfectly normal … I have no idea what it’s thinking.”* (Doctor, Lebovitz et al., 2022^17^)
			b. Strategies to promote explainability	“*I think that's a problem with some of the deep learning methods, they are abstract and that makes it very hard for a clinician to understand … ultimately the computer just pumps out a number.”* (Researcher, Hallowell et al., 2022^41^)
		iii. Evaluation and validation		*“So the AI itself, I think that trust would be established by illustrating that it worked … a good RCT showing that it worked … That just good, high quality level of evidence would do the trick for me.”* (Doctor, Hallowell et al., 2022^41^)
		iv. Clinical utility	a. Clinical impact	*“No, it may look good on paper … but the real question is, does it make any difference to what the surgeon does, or what the clinician does, or what happens to the patient?”* (Doctor, King et al., 2023^48^)
	C. Knowledge and support needed to use AI	i. Useability	a. Ease of use and learning to use the AI	“*Well in the beginning certainly there is an increase in time, because I have to learn, as you said, how the system works, learn to interpret [it], learn to believe the system, compare it[s results] with my knowledge.”* (Lombi and Rossero, 2023^49^)
			b. User interface	*“Participants demanded a self-explanatory design that can be operated quickly and in a few simple steps.”* (Buck et al., 2022^32^)
		ii. Integration with existing software		“*The lack of standard user interfaces to integrate AI results into clinical front-end software further challenges the implementation and deployment of AI in clinical practice. Suboptimal AI integration diminishes the added value of AI and hinders easy utilisation by end- users*” (Kim et al., 2024^47^)
		iii. Technical support		*“First of all, you should have low-threshold support. If it doesn't work, that you can get help immediately.”* (Doctor, Held et al., 2022^42^)
	D. Nature of data used to train the AI	i. Dataset quality	a. Dataset digitisation	*“Data are often not digitized, much is still in paper files, not structured, which means that the data availability is really extremely … poor.”* (Doctor, Pumplun et al., 2021^24^)
			b. Dataset curation and labelling	“*What if one of the radiologists who was marking … was a bit of a goofball? Now all his mistakes are being used as the template for AI …”* (Doctor, Prakash et al., 2021^54^)
			c. Representativeness of dataset	“*You want as much data as possible from as many different backgrounds as possible … I've heard statistics where like a lot of studies don't include women … so there hasn't been a lot of data on how it affects women's bodies. So I would question heavily if it wasn't more diverse.”* (Patient, Richardson et al., 2022^27^)
		ii. Dataset quantity		*“[AI] has a huge database of what diagnosis A is supposed to look like as opposed to a human who only has their own life experiences.”* (Patient, Nelson et al., 2020^16^)
		iii. Dataset over time		“*In the context of AI, one participant noted that it is important that AI tools continue to improve as additional data are available.”* (Adams et al., 2020^30^)
3. Value proposition	A. Supply-side value			*“In all cases, their research interests are grounded in a desire to answer complex questions through data, and the health care domain poses unique challenges that influence their approach.”* (Satterfield et al., 2019^59^)
	B. Demand-side value	i. Improved diagnosis	a. Benefits	“*I actually think [CT AI] is mission-critical. For me to read cases, I absolutely love having the [CT AI]. I used to not have it in my prior place [hospital]. I thought it was the worst thing ever. And then when I came here, I was amazed.*” (Doctor, Lebovitz et al., 2022^17^)
			b. Risks	*“It would be frightening, wouldn't it, you know? You know somebody could find out you've got this, you've got that, and I think it's alright as it is, you know? I wouldn't want to think that somebody could just say ‘right, we want to see you now because you've got’ [Pause] oh I don't know, some kind of cancer on top of what you've already got.”* (Patient, Winter and Carusi, 2022^65^)
		ii. Efficiency	a. Benefits	*“It can increase the productivity by 40–50% something that you do across 8 h can be now done in 4–5 h”* (Doctor, Prakash et al., 2021^54^)
			b. Risks	“*Radiologists expressed negative views of having to tediously check and ultimately, “blow off” AI results for every patient's case, especially given the high time pressure they faced: “It's not worth my time to evaluate it”* (Doctor, Lebovitz et al., 2022^17^)
		iii. Empowerment	a. Benefits	*“In the same way, some radiologists argued that they could more easily justify and communicate their assessment to colleagues, and sometimes even to the patient, if they could rely on the systems' confirmation of their own evaluation … confirming advice of the AI system bolstered these radiologists' diagnostic confidence and was seen as very desirable*.” (Jussupow et al., 2021^46^)
			b. Risks	*“As patients could feel that the AI-enabled system performs the treatment, the physicians assumed that the use of AI-enabled systems might negatively impact the physician–patient relationship.”* (Buck et al., 2022^32^)
		iv. Education	a. Benefits	*“I think it would be a great thing for learners or less experienced docs … sometimes it's helpful … for someone who has experience in that, some of the differential diagnoses are things that might not have popped into my mind.”* (Doctor, Sibbald et al., 2022^61^)
			b. Risks	“*If they were to get hacked or a system goes down … what is the contingency plan? If you have all these doctors who are so used to having this artificial intelligence read all these, and they don't have the skill of reading it, then what happens?”* (Patient, Richardson et al., 2021^26^)
		v. Equality and access to healthcare	a. Benefits	*“The effort is often associated with a lot of time for the patients … it usually takes a long time, then with the wide drop, then they are not allowed to drive themselves, then they have to organize someone.”* (Doctor, Held et al., 2022^42^)
			b. Risks	*“Is insurance only gonna cover what the machine says it is and not look for anything else? There is no reason for further diagnostics because the machine already did it? I mean we already have a situation in our healthcare system where money comes into play for diagnosing things.”* (Patient, Richardson et al., 2021^26^)
4. Adopter system	A. Staff	i. Control and trust	a. Level of control	*“[AI] would be helping to aid in the decision-making. But the human would be actually making the decision after.”* (Patient, Nelson 2020^16^)
			b. Level of trust	*“I am willing to use it, but here are two problems. If I can't always trust the ‘diagnostic decisions’ rendered by AI, then I have to double check all of its work, making it useless for me”* (Doctor, Prakash et al., 2021^54^)
		ii. Integration with diagnostic process	a. Diagnostic support	“*They perceived this as beneficial because it allowed them to be more confident in making their diagnostic decisions, knowing that their own assessments and the AI advice were well aligned, even when they were working under stressful conditions such as sleep deprivation.”* (Jussupow et al., 2021^46^)
			b. Diagnostic conflict	*“In cases where they disagreed with the system recommendation (sepsis or not), physicians reported that they would rely on their own judgment.”* (Henry et al., 2022^44^)
		iii. Changes in job role	a. Change in clinical practice	*“Another mentioned factor was that it might add to the GP's role of a mediator. Even though he may not be the one who conducts the examination, nor the one who evaluates it, he remains the one who discusses the result and its individual significance with the patient and is available for questions of understanding”* (Haan et al., 2019^40^)
			b. Existential threat	*“By the time I could get good at it both me and my knowledge could be rendered entirely and permanently obsolete by such an algorithm. I fear that we can easily become the switch board operators of medicine … I've been experiencing a serious existential fear for a while now due to this”* (Doctor, Prakash et al., 2021^54^)
		iv. Cross-disciplinary working	a. AI and clinicians	*“What can I solve myself by looking at the data and then what can I raise to the clinician to say ‘this looks kind of strange?’ So yeah and I think that's what's hugely valuable is if you can have a clinical expert to be part of the development procedure.”* (Researcher, Winter and Carusi 2022^66^)
			b. Within healthcare	*“In addition, interprofessional collaboration must be promoted in education and training. In the age of AI, physicians must work better as a team with other professional groups on an equal footing.”* (Mosch et al., 2022^51^)
	B. Patients	i. Patient choice		*“I think it all comes back to choice, though, I think everybody's getting the mentality that, and maybe I'm wrong, but that an AI is being pushed, but at the end of the day, our choice is still our choice, and it's not being taken away.”* (Patient, Richardson et al., 2021^26^)
		ii. Involvement with AI research		*“Participants expressed a desire for patients and the patient community to have a role in developing AI algorithms and collaborating on implementation.”* (Adams et al., 2020^30^)
5. Organisation	A. Capacity to innovate	i. Organisational leadership		*“Leaders and decision-makers are the key personnel who could transform AI applications in radiology work.”* MRI technician (Abuzaid et al., 2021^29^)
		ii. AI expertise		“*Although a medical background can help identify relevant training data or assess the functionality of the [AI] system, [AI] method expertise is needed to train, integrate, and operate [AI] systems … therefore, clinics need specific expertise in the field of [AI] methods in addition to their medical understanding.”* (Pumplun et al., 2021^24^)
	B. Readiness for AI	i. IT infrastructure and computing resources		*“I have some background in electronics, and one thing you can guarantee with electronics is they will fail. Might not be now, might never happen in 10, 20 years. The way things are made, ‘cause I've actually worked in the industry of making medical equipment, it's all about using the cheapest method to get the end result. Well, electronics fail. They just do.”* (Patient, Richardson et al., 2021^26^)
		ii. Stakeholder readiness	a. Healthcare professionals	*“Promoting clinician acceptance was the dominant challenge for all implementation leaders.”* (Joshi et al., 2022^45^)
			b. Patients	*“I think we can be influenced [by the patients' opinions] because, in the end, a medical practice follows the market like a small business. If the patients want [AI technologies] and demand [AI technologies], more and more practices will offer it.”* (Doctor, Buck et al., 2022^32^)
			c. Healthcare leaders	*“We've got a workforce crisis so I'll tell you that the reason that AI is gaining support from Public Health England and gaining momentum is because of the workforce crisis.”* (Doctor, Chen et al., 2021^35^)
	C. Nature of adoption or funding decision	i. Cost of AI		“*So, this is a really complex question to answer and, essentially, if we were to follow … guidelines, we basically have to show a health economic benefit within* 12 months*. And my feeling is that we may not demonstrate [regulatory body] gold standard of health economic benefits in* 12 months*, particularly given how expensive it is to get everything into one place.”* (Senior manager, Faric et al., 2023^37^)
	D. Extent of change needed to routines	i. Care pathways within organisations		*“Various aspects were mentioned here that must be discussed with the entire practice team. It must be clarified at what time, at what time interval, in which room and by whom the examination [with AI] will be carried out.”* (Held et al., 2022^42^)
		ii. Care pathways between organisations		*“Dermatologists and GPs expected the use of AI to facilitate substitution of low-risk skin cancer care (e.g., low-risk basal cell carcinomas, actinic keratosis) from the dermatologist to the GP practice.”* (Sangers et al., 2023^18^)
	E. Work needed to implement change	i. Implementation strategy		*“As [AI] systems are a strategically relevant innovation, not only is the support of the directors necessary but also the establishment of an overarching, long-term [AI] strategy.”* (Pumplun et al., 2021^24^)
6. Wider context	A. Political/policy context			*“They considered assurance of the quality of mHealth [AI] apps to be a matter of government regulation.”* (Sangers et al., 2021^58^)
	B. Regulatory/legal	i. Regulatory framework		*“AI that the dermatologist uses … should really be tested by an authoritative organization with independent research, at least two preferably. So just like you register a drug.”* (Doctor, Sangers et al., 2023^18^)
		ii. Data access, privacy, and security		*“I was just gonna say another concern … is can that artificial intelligence be hacked … I don't care what anybody says, it can and it will get hacked because there's always somebody that's out there just to do evil rather than good.”* (Patient, Richardson et al., 2021^26^)
		iii. Liability		*“It has to be the human's responsibility because AI is just an aid, it's like a piece of software. You know you can't hold it responsible.”* (Patient, Winter and Carusi, 2022^65^)
	C. Professional bodies	i. Medical professional bodies		*“Experts see medical professional associations as having a responsibility to develop positions and concepts for the introduction of AI in medical practice, education, and research.”* (Mosch et al., 2022^51^)
		ii. AI developers/researchers		*“Like who decides what's good or bad? It's relative depending on whatever company wants to make a bunch of money off their data. That's what I'm the most nervous is about the corporate side of it.”* (Patient, Richardson et al., 2022^27^)
	D. Socio-cultural	i. Attitudes towards and experiences of technology	a. Broader technology	*“Unless you're like my uncle, my uncle says that we'd all be better off if we went back to the times where all this technology hadn't been invented and computers hadn't been invented. He says computers are a fad.”* (Patient, Richardson et al., 2022^27^)
			b. AI	*“I know it's a movie, but I mean there have been movies about intelligence becoming intelligent enough to figure out how emotion works … and then computers take over computers, and … humans become completely obsolete.”* (Patient, Richardson et al., 2022^27^)
		ii. Experiences of healthcare systems		*“Participants who were more optimistic about healthcare AI, by contrast, described long and demanding diagnostic journeys involving repetitive testing or multiple visits to specialists.”* (Richardson et al., 2022^27^)

### Domain 1: Condition

Stakeholders discussed the situated nature of diagnostic AI, considering the context and scope of implementation. Clinicians emphasised the importance of human control of when, where, and how AI is used in the diagnostic pathway.

#### 1A: Nature of condition/task

Clinicians and researchers believed that AI should handle administrative tasks,^34^^,^^49^^,^^52^^,^^62^^,^^63^ synthesise medical information,^16^^,^^59^ and perform image processing.^36^^,^^42^^,^^52^ Appropriate image processing tasks included pre-screening images for triage,^36^^,^^42^^,^^48^^,^^49^^,^^52^^,^^55^^,^^58^^,^^60^ or quantifying regions of interest within medical images.^36^^,^^48^

Some viewed AI as a source of reassurance in diagnosing rare diseases, grounded in the perception that AI “*work(s) with a larger database than the human brain”*.^32^^,^^34^^,^^66^ Others believed AI could facilitate longitudinal disease review by integrating self- and clinician-assessment.^18^^,^^49^^,^^58^

#### 1B: Clinical context

Clinicians discussed the suitability of AI in different clinical settings. Many believed that currently, AI's effectiveness was confined to well-defined, narrow applications, limiting its utility.^24^^,^^43^^,^^47^ Incorporation of contextual information, such as patient co-morbidities,^24^^,^^26^ multi-modal data,^36^ clinical examination^16^^,^^18^^,^^24^^,^^41^^,^^44^ and non-verbal patient cues^32^ were simultaneously perceived to be of paramount importance and beyond the scope of AI. A clinician explains, “*I don't think you can teach a machine how to interpret clinical context, it's too nebulous*”.^48^ These views were mirrored by patients, who discussed the importance of context gained from in-person appointments and knowledge of patient clinical history.^26^^,^^58^

#### 1C: Nature of setting

Clinicians and leaders noted a discrepancy between settings which would benefit most from AI, and resource constraints in such settings for its implementation.^24^^,^^42^^,^^48^ AI was seen as more beneficial for clinicians working in less specialised or rural facilities, compared to well-resourced centres.^42^^,^^48^ Some emphasised the importance of local control of AI implementation: “*blanket introduction from on high is likely to raise people's suspicions”*.^48^

### Domain 2: Technology

The features of AI were perceived as crucial determinants of trustworthiness and useability. Patients and clinicians viewed qualities such as high accuracy, empirical validation of results, and representative training data as enhancing trust in AI. However, trustworthiness alone was not sufficient: human control was described as necessary for AI adoption.

#### 2A: Material features

All stakeholders felt that AI lacks essential human qualities such as compassion, empathy, and intuition: *“you can't teach an algorithm to love somebody”*.^16^^,^^30^^,^^32^, ^33^, ^34^^,^^37^^,^^38^^,^^40^^,^^53^^,^^57^^,^^62^ Emphasis was placed on non-verbal cues and physical interaction.^16^^,^^32^, ^33^, ^34^^,^^38^^,^^40^^,^^52^^,^^62^ A patient stressed the importance of, “*human touch and the human eye contact*”,^16^ and echoed by a clinician: “*[patients] want to be touched … to look you in the eyes”*.^32^

Patients expressed the desire to be treated as individuals.^38^^,^^40^^,^^41^^,^^57^ They valued the capacity to ask questions, discuss results, and seek reassurance, underscoring a preference for humans.^16^^,^^26^^,^^33^^,^^34^^,^^40^^,^^58^ Human intuition was considered by some clinicians to be important for accurate diagnosis, and not replicable by AI: *“There is a lot of what is called ‘gut feeling’ involved … sometimes you just look at the person and you can tell they are really sick”*.^32^^,^^34^^,^^62^

#### 2B: Type of data generated

##### Quality of diagnosis

All groups viewed high diagnostic accuracy as essential, although opinions varied on minimal acceptable levels of accuracy.^16^^,^^18^^,^^30^^,^^33^^,^^35^^,^^42^^,^^50^^,^^52^^,^^56^^,^^58^^,^^61^ Some asserted that AI should work “*flawlessly*”,^58^ or face redundancy.^18^^,^^32^^,^^33^^,^^48^^,^^54^^,^^58^ Researchers, however, were suspicious of very high accuracy: “*[If] it's really good I'm generally filled with doom”*.^66^

Clinicians and researchers linked diagnostic quality with AI's perceived credibility.^17^^,^^41^^,^^43^^,^^50^^,^^59^^,^^61^^,^^65^^,^^66^ For many, credibility was measured by correlation with clinician opinion.^41^^,^^46^^,^^61^^,^^65^^,^^66^ A researcher reflected, “*clinicians might come to regard [AI] tools as useful, if they produce results that agree with them”*.^41^ This was reflected by assertions of clinicians rejecting AI: “*I do not need feedback from a software … it is rarely right”*.^46^

##### Explainability and transparency

Explainability of AI results is seen as crucial, particularly when they diverge from clinician judgements.^17^^,^^18^^,^^24^^,^^31^^,^^32^^,^^36^^,^^38^^,^^39^^,^^41^^,^^42^^,^^44^, ^45^, ^46^^,^^48^, ^49^, ^50^^,^^54^^,^^56^^,^^59^, ^60^, ^61^, ^62^, ^63^^,^^65^^,^^66^ When divergent AI results were not explainable, clinicians expressed frustration: “*How is it coming up with this … How does it know?“*.^17^

Some highlighted the gestalt nature of clinical diagnosis. A researcher described clinicians as “*black boxes too … they can hardly explain … [their] diagnosis”*.^41^ Some clinicians agreed: *“We work in a complete black box; they don't question us”,* suggesting that prioritising explainability may limit AI's capabilities.^48^

Researchers described the difficulty of making AI predictions comprehensible to humans: *“[AI]-based diagnostics are probabilistic … not based on anatomical features”*.^41^ Strategies such as *“relatable metaphors”*^45^ and image heatmaps^55^^,^^65^^,^^66^ were suggested as ways to improve explainability.

##### Evaluation and validation

For some, AI evaluation and validation supersedes explainability.^36^ Clinical trials, government regulation, post-production evaluation, and peer review were all cited as methods to demonstrate trustworthiness.^18^^,^^30^^,^^32^^,^^33^^,^^36^^,^^40^, ^41^, ^42^^,^^44^^,^^45^^,^^48^^,^^51^^,^^54^^,^^60^^,^^61^^,^^63^ Clinicians, leaders, and researchers described the importance of local validation and configuration for diagnostic AI: this adjustment of AI parameters such as sensitivity/specificity to account for the demographics of the local patient population.^31^^,^^37^^,^^47^

##### Clinical utility

Participants also require AI to demonstrate clinical utility through actionable results, and clear evidence of patient benefit.^18^^,^^36^^,^^42^^,^^45^^,^^46^^,^^48^^,^^50^^,^^62^^,^^66^

#### 2C: Knowledge and support

All stakeholders shared frustration with existing *“complicated and cluttered”* computer systems, considering AI an opportunity for improvement.^16^^,^^18^^,^^24^^,^^25^^,^^32^^,^^38^^,^^42^^,^^45^^,^^48^^,^^60^ AI tools must be fast, user-friendly, and have accessible technical support for adoption by time-pressured clinicians.^18^^,^^25^^,^^31^^,^^32^^,^^37^^,^^38^^,^^42^, ^43^, ^44^, ^45^^,^^47^, ^48^, ^49^^,^^58^^,^^60^^,^^62^^,^^64^

#### 2D: Nature of data used to train/test AI

All groups linked AI's perceived trustworthiness to training/test data volume and quality. Medical data is viewed as plentiful, but researchers and clinicians describe its unstructured, idiosyncratic nature as hindering AI development.^24^^,^^31^^,^^36^^,^^39^^,^^51^ Patients and clinicians expressed doubts about the accuracy of medical data and resulting AI validity: *“we all know how many mistakes there are in medical records”*.^26^^,^^63^

Stakeholders emphasised the importance of representative datasets, to minimise the risk of embedded racial and sex bias.^18^^,^^24^^,^^26^^,^^27^^,^^33^^,^^44^^,^^52^^,^^56^^,^^66^ For some, even if an AI pipeline has not been shown to be biased, the *“perception of bias can be just as damaging”* if a demographic has not been represented in training data.^56^ Participants expressed that AI should be periodically retrained, in-line with technological advances, changes in patient demographics, and clinical context.^17^^,^^18^^,^^24^^,^^30^^,^^59^

Data curation and labelling to establish reference standards was considered subjective and flawed.^36^^,^^54^ Proposed solutions included standardising medical records, referencing structured data, or using multi-disciplinary team decisions.^24^^,^^66^

### Domain 3: Value proposition

All stakeholder groups specified that AI must add value compared to usual practice for sustained adoption. This was largely represented by demand-side value, for patients, clinicians, and healthcare services. However, for each representation of value, stakeholders identified associated risks.

#### 3B: Demand-side value

##### Diagnosis

All groups perceived AI as improving diagnostic accuracy and consistency, through processing vast quantities of data.^16^, ^17^, ^18^^,^^24^, ^25^, ^26^, ^27^^,^^29^^,^^32^^,^^36^^,^^45^^,^^51^^,^^52^^,^^54^^,^^55^^,^^59^^,^^63^ Researchers believed that AI could exceed human capacity for data analysis and facilitate precision medicine.^24^^,^^36^^,^^51^^,^^59^^,^^66^

AI is perceived as objective, consistent, and indefatigable, and therefore less prone to error.^16^, ^17^, ^18^^,^^25^^,^^32^^,^^36^^,^^41^^,^^55^ One clinician viewed AI as safeguarding against incompetent practitioners: a tool to “*protect people against themselves”*.^63^

Concerns arose regarding AI errors stemming from flawed training datasets.^24^^,^^40^^,^^54^^,^^58^^,^^65^ Some clinicians expressed apprehension about the potential for AI-driven overdiagnosis and overtreatment.^32^^,^^38^^,^^44^^,^^54^ This was mirrored by some patients, feeling that AI could cause unnecessary anxiety through *“learning too much about one's health”*.^40^^,^^65^

##### Efficiency

AI was perceived as improving efficiency through reducing image acquisition times, and automating triage and administrative tasks without fatiguing.^18^^,^^24^^,^^25^^,^^27^^,^^30^^,^^32^, ^33^, ^34^^,^^36^, ^37^, ^38^, ^39^, ^40^^,^^42^^,^^43^^,^^49^^,^^51^^,^^52^^,^^54^^,^^56^^,^^60^^,^^62^^,^^64^^,^^65^ All groups believed AI could relieve clinician workload, allowing them to concentrate on complex tasks and fostering patient relationships: “*the “real” craft of medicine”*.^25^^,^^32^^,^^34^, ^35^, ^36^^,^^38^^,^^42^^,^^44^^,^^49^^,^^51^^,^^52^^,^^54^^,^^56^^,^^60^^,^^62^ Patients framed efficiency using personal experiences of diagnostic delay and considered improved efficiency as lifesaving.^16^^,^^27^^,^^30^^,^^33^

However, some clinicians worried that AI might reduce efficiency, adding unnecessary diagnostic steps.^17^^,^^32^^,^^45^^,^^49^^,^^63^ Others believed that AI would, *“leave them with just the difficult cases, increasing work intensity”*.^48^

##### Empowerment

Some clinicians perceived AI as empowering, fostering confidence in diagnostic decisions.^17^^,^^37^^,^^43^^,^^46^^,^^64^ An ethnographic study found that even when clinicians disagreed with AI results, their confidence increased.^17^ Similarly, patients considered AI as democratising medical information, enabling self-assessment, and informing healthcare choices.^16^^,^^30^^,^^33^^,^^34^^,^^58^ Some researchers considered AI as a tool to recalibrate the power balance between patient and doctor: “*Doctors need to get off their pedestals, and the patients need to get off their knees”*.^39^

Other clinicians feared that AI could promote over-confidence in less experienced or less specialised colleagues.^34^^,^^42^^,^^49^^,^^54^ One radiologist described this as creating the risk of, “*DIY-radiologist[s] [with AI]”*.^49^ Conversely, some felt AI could disempower clinicians by assuming the diagnostic role.^32^^,^^54^ Patients worry that AI might *“distract the attention of providers to their computers”*,^27^ harming the doctor-patient relationship.^32^^,^^42^

##### Education

Some clinicians view AI as an educational tool, particularly if AI provided feedback on decision-making.^18^^,^^32^^,^^36^^,^^41^, ^42^, ^43^^,^^46^^,^^61^^,^^63^ Others believed that AI would be helpful for less experienced clinicians but denied personal benefit: *“I am chief physician … I don't allow it to influence my decision”*.^36^^,^^39^^,^^46^^,^^61^^,^^63^

However, clinicians and patients expressed concern regarding automation bias and deskilling, particularly in the event of AI failure.^16^^,^^18^^,^^26^^,^^32^^,^^34^^,^^35^^,^^37^^,^^38^^,^^41^^,^^44^^,^^48^^,^^49^^,^^51^^,^^54^^,^^56^^,^^61^, ^62^, ^63^, ^64^, ^65^ Novice practitioners were perceived as especially susceptible to AI overreliance, leading to concerns about the future medical workforce.^18^^,^^33^^,^^38^^,^^41^^,^^54^^,^^56^^,^^64^^,^^65^

##### Equality

Clinicians and patients viewed AI as broadening healthcare access for underserved populations through remote diagnosis.^16^^,^^27^^,^^30^^,^^31^^,^^34^^,^^42^ Improved efficiency, streamlined referrals, and personalised treatment recommendations were anticipated to reduce healthcare cost.^16^^,^^34^^,^^40^ One clinician took the view that referrals, “*make the ophthalmologists' cash register ring … we don't want that anymore”*.^42^

However, some patients feared an increase to their personal costs, either as a direct result of paying for AI, or indirectly through increased insurance premia: *“get your chequebook out”*.^26^^,^^33^^,^^58^^,^^65^ Others feared that treatment options would be restricted by AI recommendations.^26^^,^^50^

### Domain 4: Adopter system

Clinicians and patients were identified as key stakeholder groups whose roles would change with AI adoption. Stakeholders once again emphasised the importance of human control at both individual and societal levels.

#### 4A: Staff

##### Control and trust

All groups agreed that humans should retain control over AI.^16^^,^^30^^,^^32^, ^33^, ^34^^,^^36^, ^37^, ^38^, ^39^, ^40^, ^41^, ^42^, ^43^, ^44^^,^^46^, ^47^, ^48^, ^49^, ^50^, ^51^^,^^53^^,^^54^^,^^56^^,^^57^^,^^59^^,^^62^, ^63^, ^64^ This was related to inherent trust in human abilities, compared to scepticism of AI. Patients stressed the importance of human control: “*it's [AI] used as a tool … as opposed to being used instead of a dermatologist”*.^16^ Likewise, researchers and clinicians viewed AI as a supplementary tool and felt that clinicians should have discretion to override it.^17^^,^^30^^,^^32^^,^^33^^,^^36^, ^37^, ^38^^,^^40^, ^41^, ^42^^,^^44^^,^^46^, ^47^, ^48^, ^49^, ^50^, ^51^^,^^53^^,^^56^^,^^57^^,^^59^^,^^62^^,^^63^^,^^65^ Clinicians and patients expressed a preference for human judgement over divergent AI findings.^16^^,^^34^^,^^37^^,^^43^^,^^44^^,^^49^^,^^53^ For some, this results in total rejection, *“blowing [it] off”*, of the AI result.^17^

Building trust in AI was viewed as incremental, through gradual integration within clinical environments.^31^^,^^37^^,^^38^^,^^41^, ^42^, ^43^^,^^45^^,^^47^^,^^48^^,^^59^^,^^60^^,^^62^^,^^65^ One researcher commented, “*You can't just put a computer in a room and say, ‘Right, you need to trust it now’”*^41^ Clinicians felt that access to AI tools over time would increase trust, through normalising its use and demonstration of benefit over time.^37^^,^^41^^,^^43^^,^^44^^,^^46^^,^^52^^,^^60^^,^^62^^,^^63^

##### Changes to job role

All groups felt that diagnostic AI would change the roles of clinicians. Some advocated AI literacy as a core job competency: “*AI won't replace radiologists, but radiologists who use AI … will replace radiologists who don't”*.^25^^,^^29^^,^^30^^,^^32^^,^^35^, ^36^, ^37^^,^^49^^,^^51^^,^^52^^,^^65^ Clinicians were seen as “*overseers”* and intermediaries between patients and AI, with their endorsement crucial for patient trust.^30^^,^^33^^,^^34^^,^^37^, ^38^, ^39^, ^40^, ^41^, ^42^, ^43^^,^^47^^,^^49^^,^^51^^,^^56^^,^^64^

Some clinicians perceived AI as a professional threat, leading to “*a sense of rivalry between human and machine”*.^18^^,^^24^^,^^29^^,^^32^^,^^46^^,^^52^^,^^54^ Others remained unthreatened, citing their work's complexity or physicality as beyond AI's scope, attributing colleagues' concerns to vanity or low self-esteem: *“I don't see software as my antagonist, I see it as my* support*”*.^25^^,^^32^^,^^35^^,^^42^^,^^49^^,^^51^^,^^52^^,^^63^

Many viewed the emergence of AI as an opportunity to shape their profession, through defining AI applications, performance review, and steering implementation.^18^^,^^25^^,^^29^^,^^35^^,^^37^^,^^45^^,^^48^^,^^51^^,^^54^ Healthcare leaders asserted that clinicians must lead AI implementation to *“fulfil their responsibility”*.^51^ Researchers described cross-disciplinary collaboration as *“priceless”* to foster trust.^38^^,^^39^^,^^47^^,^^49^^,^^65^^,^^66^

#### 4B: Patients

Patients emphasised the importance of autonomy: *“our choice is still our choice, and it's not being taken away”*.^26^ This included the choice to have AI tools used or excluded from their care, whether, and when to have clinician review.^26^^,^^33^^,^^38^^,^^57^^,^^58^^,^^65^ Some believed patients should participate in AI development to ensure responsible use.^30^

### Domain 5: Organisation

All stakeholder groups considered the mechanisms required for smooth integration of AI at an organisational level. Healthcare leaders in particular considered strategies for scaling-up and spreading AI use across organisations.

#### 5B: Readiness for AI

Healthcare leaders and researchers viewed clinicians as gatekeepers for implementation.^31^^,^^34^^,^^37^^,^^45^^,^^47^^,^^56^ Conversely, clinicians described the influence of other stakeholder groups; patients, politicians, and employing institutions as the driving force behind AI adoption.^25^^,^^32^^,^^35^^,^^45^^,^^54^

Existing workplace IT infrastructure was often regarded as inadequate for AI implementation, and prone to failure.^26^^,^^32^^,^^36^^,^^38^^,^^39^^,^^48^^,^^64^ Organisations often work with multiple AI providers, each with different applications and user interfaces.^17^^,^^37^^,^^47^ One study found that the implementation of a ‘*vendor-neutral AI platform’*, to which all potential AI providers adapt their software, reduced downstream costs, and streamlined adoption.^47^

#### 5C: Nature of adoption or funding decision

Clinicians and healthcare leaders highlighted *“health economic benefit”* as key to sustained AI adoption, particularly in competing with other healthcare interventions.^18^^,^^24^^,^^25^^,^^32^^,^^36^, ^37^, ^38^^,^^42^^,^^45^^,^^47^^,^^62^ Some clinicians worried that individual practices would need to fund AI development or procurement.^25^^,^^32^^,^^36^^,^^42^^,^^45^^,^^48^ Others were wary of commercial exploitation: *“we don't profit … the large corporation somehow gets 50 euros per photo”*.^42^

#### 5D: Extent of change needed to routines

All stakeholder groups highlighted the importance of clear AI guidance within and between organisations.^18^^,^^24^^,^^25^^,^^42^^,^^43^^,^^46^, ^47^, ^48^^,^^51^^,^^58^^,^^60^^,^^65^ Clinicians expected AI to improve interactions across care settings.^18^^,^^42^^,^^58^^,^^60^^,^^65^ Some patients and clinicians felt the value of diagnostic AI lay in facilitating direct contact with specialist providers, bypassing non-specialists.^58^^,^^65^

### Domain 6: Wider context

In this domain, stakeholders discussed the obligations of legal and professional bodies to establish clear frameworks for implementation, considering this essential to trust the widespread use of AI.

#### 6B: Regulatory/legal

All stakeholder groups emphasised the importance of data privacy and security due to the sensitivity of medical data.^16^^,^^18^^,^^24^^,^^26^^,^^30^, ^31^, ^32^, ^33^^,^^37^, ^38^, ^39^^,^^42^^,^^56^^,^^58^^,^^62^^,^^65^^,^^66^ Concerns included potential data misuse by “*nefarious”*^16^^,^^32^ actors: *“somebody that's out … to do evil”*.^26^ Linked to these concerns was a preference for data anonymisation and minimal data collection.^30^^,^^58^ A robust regulatory framework was a pre-requisite for use.^18^^,^^24^, ^25^, ^26^^,^^37^^,^^38^^,^^51^^,^^54^^,^^56^^,^^59^ However, some cautioned against *“AI exceptionalism”*, in which AI technologies are held to a prohibitively high standard, compared to other healthcare interventions.^56^ Researchers, leaders, and clinicians describe the requirement for data sharing between organisations to develop and validate diagnostic AI, and some consider current information governance restrictions to be too restrictive.^31^^,^^37^^,^^38^^,^^56^

Legal liability for diagnostic errors was a common concern. For many, it was clear that clinicians would retain liability, providing patients with *“someone to sue”* in the event of error.^17^^,^^33^^,^^36^^,^^40^^,^^42^^,^^49^^,^^52^^,^^54^^,^^64^, ^65^, ^66^ This was a source of anxiety for some clinicians,^17^^,^^18^^,^^25^^,^^42^^,^^48^^,^^52^^,^^61^ illustrated by a radiographer who worried, “*When things go wrong … do I blame the robot, or can I blame the hospital”*.^52^ Others believed clinicians and AI developers should share responsibility for AI errors, protecting individual practitioners.^16^^,^^32^^,^^35^ Clinicians in one study made a distinction between diagnostic discrepancy, which was felt to be acceptable differences in professional opinion, and error, a *‘failure to meet expected standards’*.^56^ It was unclear, in the case of AI, *“how much discrepancy amounts to error and whether a standard needs to be set for hybrid pathways”*.^56^

#### 6C: Professional

Patients and clinicians expressed distrust of AI developed by commercial entities, worrying that companies may lie about, or embellish their results; “*putting lipstick on a pig”*.^16^^,^^18^^,^^27^^,^^32^^,^^33^^,^^41^^,^^42^^,^^44^^,^^45^^,^^58^^,^^65^ Many held a preference for AI developed by clinicians or research institutions.^16^^,^^17^^,^^45^^,^^58^^,^^65^ Some clinicians worried about reputational damage through commercial associations: *“I don't want them to think my agenda is a company that I am working with”*.^41^

For some, regulation,^27^ or evidence of *“public benefit sharing”*^41^ would be sufficient to overcome distrust of commercial companies. Clinicians expected professional organisations to establish frameworks for AI use and felt this was necessary to trust individual softwares.^18^^,^^32^^,^^44^^,^^48^^,^^51^ Others made a distinction between trust, and *“trust-like”* behaviour.^41^ If AI use is widespread, clinicians may engage with it despite not trusting it, “*because they are locked into a dependency relationship [with it]”*.^41^

#### Conceptual model

**Fig. 2:**
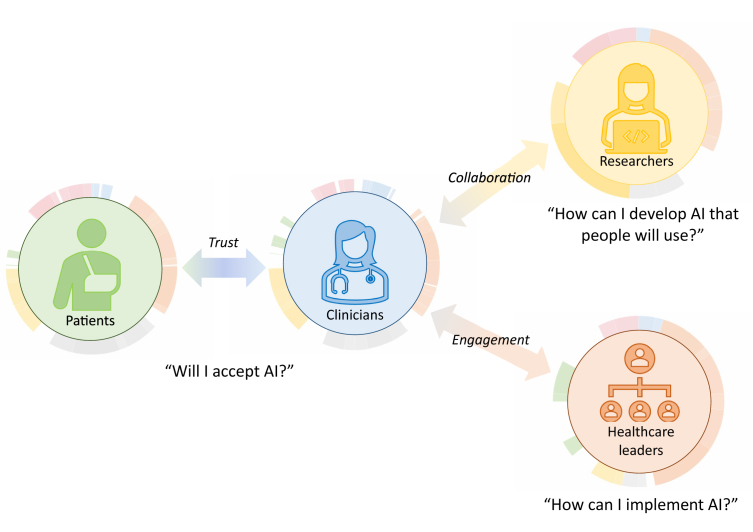
Visual representation of a conceptual model of stakeholder groups and relationships.

We identified four stakeholder groups: patients, clinicians, researchers, and healthcare leaders. Each group placed emphasis on different domains, subdomains, and themes of the NASSS framework. In comparing the perspectives of each group, we found they had different key questions and concerns, and relationships between groups were governed by distinct principles. We have developed a conceptual model to visualise these relationships and perspectives (Fig. 2).

The primary question for patients and clinicians was, “Will I accept AI?”. For patients, determinants to the question centred around individual risk and benefit, and feelings of control. Positive determinants included AI's potential to improve diagnostic accuracy (Domain 2B) and efficiency (3D), informed by prior negative experiences with existing healthcare systems (6D). Negative determinants were loss of control over their data to commercial companies (6B/C), reduced choice due to increased personal cost (3B) and increasing social inequalities (3B). For patients, the human aspect of diagnosis was crucial (2A). Patients placed high value on clinician interaction, and acceptance of diagnostic AI hinged on trust within this relationship (2A/4A). Likewise, clinicians recognised the importance of their relationship with patients (2A/4A).

For clinicians, determinants to this question related to feelings of autonomy, control, and safety. Clinicians favoured high levels of control: where, when, and how AI is deployed (1), the ability to interrogate and potentially reject datasets, algorithms, and results (2B/C/D/4A). AI that confirmed clinician opinion (3B/4A), acted as a safeguard for clinician error (3B), or was endorsed by professional bodies (6C), contributed to feelings of professional safety. Negative determinants were situations where clinicians had low levels of control and safety–for example, the imposition of AI (1/5), taking full liability for AI errors (6B) or the threat of replacement (4A). “Top-down” imposition of diagnostic AI by healthcare leaders was associated with loss of control (1/5), whilst collaboration with researchers in proposing, developing, and implementing AI contributed increased feelings of autonomy (4A).

Researchers expressed high levels of confidence in the material features, and added value of AI: improved diagnostic accuracy, consistency, and increased efficiency of clinical workflows (2/3). Their key question was, “How can I develop AI that people will use?”. This was seen as dependent on clinician engagement (4A/5B). They placed emphasis on a collaborative relationship with clinicians throughout AI development, valuing their input in understanding medical data and evaluating outputs (4A/5B).

Healthcare leaders regarded AI as a means to make clinical workflows more efficient and reduce diagnostic error (2/3B). As with researchers, they viewed clinicians as crucial in determining AI adoption (5B). Their key question was, “How can I implement AI?, emphasising clinician engagement. This centred around processes for smooth integration (2C/4A/5), including aspects of the technology: technical support, useability and explainability, and integration with existing systems (2B/C/D). The other dimensions considered by healthcare leaders were the logistical aspects of AI integration within the organisation, with particular emphasis on the clinician “readiness” for AI (5/6).

## Discussion

In this QES, we synthesise current qualitative work on stakeholder perceptions of diagnostic AI, and present the NASSS-AI framework. The NASSS-AI framework is an extension of the NASSS framework with deeper granularity, tailored specifically for the implementation of diagnostic AI. We have introduced two AI-specific subdomains, and 43 themes to better inform future implementation strategies. For example, we found that all stakeholder groups place importance on the provenance, accuracy, and representativeness of health data used to train and test AI. This suggests that clear records of training and test metadata, and granular reporting of AI performance by demographic group, would improve perceived trustworthiness of individual software. The need for robust regulatory frameworks establishing best practice for data privacy, security and legal liability were discussed by all stakeholder groups, highlighting the importance of legal sector and government involvement at a societal level. Using the NASSS-AI framework, we developed a conceptual model to describe the perspectives of, and relationships between patients, clinicians, researchers, and healthcare leaders, situated in the data. For example, a crucial relationship dynamic between clinicians and leaders, is the level of engagement between these stakeholder groups. Healthcare leaders seeking clinician engagement with diagnostic AI could employ implementation strategies that have explicit elements of clinician control: an important determinant for acceptance in this stakeholder group.

This review has some limitations. First, we included only English-language articles, which may introduce cultural bias to our results. Although there was a diverse geographical spread of publications, we found an over-representation of studies originating from high-income countries, and some regions were not represented in our data. However, performing the electronic searches without language restriction did not significantly change the number of retrieved results (<2%). Second, there was limited information about the demographics of study participants. Only 23 reported participant sex,^16^^,^^18^^,^^26^^,^^27^^,^^30^, ^31^, ^32^, ^33^, ^34^^,^^38^^,^^40^^,^^42^^,^^48^^,^^49^^,^^52^, ^53^, ^54^^,^^57^^,^^58^^,^^60^, ^61^, ^62^, ^63^^,^^67^ eighteen age,^16^^,^^18^^,^^26^^,^^27^^,^^31^^,^^33^^,^^34^^,^^37^^,^^38^^,^^40^^,^^42^^,^^49^^,^^52^, ^53^, ^54^^,^^57^^,^^58^^,^^61^^,^^62^ and five ethnicity.^16^^,^^26^^,^^27^^,^^38^^,^^53^^,^^64^ Sixteen studies employed convenience or snowball sampling for participant recruitment, and may have introduced selection bias, limiting transferability of their findings.^26^^,^^27^^,^^29^^,^^32^^,^^34^, ^35^, ^36^, ^37^, ^38^^,^^41^^,^^42^^,^^45^^,^^46^^,^^53^^,^^57^^,^^64^ Third, of the included stakeholder groups, patients, researchers, and healthcare leaders were under-represented compared to clinicians. Only thirteen explored patient views,^26^^,^^27^^,^^30^^,^^40^^,^^58^^,^^65^^,^^66^ nine studied those in leadership positions,^24^^,^^25^^,^^31^^,^^34^^,^^37^^,^^39^^,^^45^^,^^47^^,^^56^ and ten studied researchers,^24^^,^^36^^,^^38^^,^^39^^,^^45^^,^^51^^,^^56^^,^^59^^,^^65^^,^^66^ compared to 26 that studied doctors’ opinions.^17^^,^^18^^,^^24^^,^^25^^,^^31^^,^^32^^,^^34^, ^35^, ^36^^,^^38^^,^^39^^,^^41^^,^^42^^,^^44^^,^^46^, ^47^, ^48^^,^^55^^,^^56^^,^^59^, ^60^, ^61^^,^^63^^,^^65^, ^66^, ^67^ We would recommend future qualitative research exploring the perspectives of under-represented stakeholder groups.

Fourth, 31 studies considered the hypothetical use of AI,^16^^,^^18^^,^^24^, ^25^, ^26^, ^27^^,^^29^, ^30^, ^31^, ^32^, ^33^, ^34^, ^35^, ^36^^,^^38^, ^39^, ^40^, ^41^, ^42^, ^43^^,^^48^, ^49^, ^50^, ^51^, ^52^^,^^54^^,^^56^^,^^58^^,^^59^^,^^63^^,^^65^ and only thirteen discussed AI tools in use.^17^^,^^37^^,^^38^^,^^44^, ^45^, ^46^, ^47^^,^^55^^,^^57^^,^^60^^,^^61^^,^^64^^,^^66^ Participants with real-world experience of diagnostic AI may have different perspectives and priorities. However, we found that there were many commonalities between participants with and without direct experience of AI. Furthermore, as diagnostic AI is not currently widely implemented, most stakeholders are “AI-naïve”, and our insights will be particularly relevant for them. Finally, our review focused directly on diagnostic AI, rather than other applications of clinical AI, such as health chatbots, or AI for research purposes.

Our review agrees with and validates the findings of Young et al., (2021),^68^ a mixed-methods review of patient attitudes towards clinical AI. In common with our review, they concluded that key concerns for patients included maintaining humanity in clinical interactions, the perceived personal risks and benefits of AI accuracy and errors, and healthcare access and inequality. We build on their findings by describing the views of the other key stakeholder groups and identify areas of under-representation.

In conclusion, we systematically reviewed qualitative research exploring multi-stakeholder perspectives on the sustained implementation of AI. We mapped this to the NASSS framework, and present an extended NASSS-AI framework tailored to implementation of diagnostic AI. Our review group has representation from clinicians, researchers, and PPI contributors. Each author held a different viewpoint, and through collaborative working we reached a rich understanding of the complexities and nuances of stakeholder perspectives. We present a conceptual model describing the different priorities of each stakeholder group in adoption of diagnostic AI, and the key aspects of between-group relationships. The NASSS-AI framework and the stakeholder conceptual model may be taken in tandem to inform future implementation strategies for diagnostic AI.

## Contributors

RK conceptualized the research, recruited and trained PPI collaborators, developed search strategies, performed screening, accessed and verified the data, data extraction, framework analysis, data visualisation, and wrote, reviewed and edited the manuscript. AF conceptualized the research, trained PPI collaborators, developed search strategies, performed screening, accessed and verified the data, data extraction and analysis, data visualisation, and reviewed and edited the manuscript. JS conceptualized the research, performed screening, data extraction and analysis, data visualisation, and reviewed and edited the manuscript. RH performed screening, data extraction and analysis, accessed and verified the data, data visualisation, and reviewed and edited the manuscript. JC performed screening, data extraction, accessed and verified the data, data analysis, data visualisation, and reviewed and edited the manuscript. DJ performed screening, accessed and verified the data, data extraction and analysis, and reviewed and edited the manuscript. EH conceptualized the research, developed and performed search strategies, and reviewed and edited the manuscript. GC conceptualized the research, advised on search strategies, framework selection and reporting guidance, supervised the methodology and reviewed and edited the manuscript. ET conceptualized the research, advised on search strategies, framework selection and reporting guidance, supervised the methodology and data analysis, accessed and verified the data, performed data analysis, and reviewed and edited the manuscript. DF conceptualized the research, advised on search strategies, framework selection and reporting guidance, supervised the methodology and data analysis, and reviewed and edited the manuscript.

## Data sharing statement

Data are presented in the manuscript and appendices. Further data that supports the findings of this study are available from the corresponding authors on reasonable request.

## Declaration of interests

RK is supported by an NIHR Doctoral Research Fellowship grant (NIHR302562), the British Society for Surgery of the Hand (BSSH), the Royal College of Surgeons of England and the Oxfordshire Health Services Research Committee. None of these organisations have had input into study design, data analysis or interpretation.
