# Supplementary material for: Stakeholder perspectives towards diagnostic artificial intelligence: a co-produced qualitative evidence synthesis
Source: eClinicalMedicine. 2024 Mar 22;71:102555. doi: 10.1016/j.eclinm.2024.102555 (PMC10973718; doi:10.1016/j.eclinm.2024.102555)
Supplement: Supplementary Material 2 [file mmc1.docx]

## NDORMS: Rachel Kuo

Rachel Kuo, Dominic Furniss, Gary Collins, Elizabeth Tutton, Eli Harriss, Judi Smith, Rosie Hill, Joanna C, Derek J, Alexander Freethy. **Perspectives of healthcare professionals and patients on the factors influencing the adoption of diagnostic artificial intelligence in clinical practice: a systematic meta-synthesis of qualitative studie**s. PROSPERO 2022 CRD42022313782 Available from: <https://www.crd.york.ac.uk/prospero/display_record.php?ID=CRD42022313782>

Eli Harriss (Bodleian Health Care Libraries, University of Oxford, ORCID: 0000-0003-4635-8959) developed the search strategy and syntax, advised on the databases to use, ran and then updated the searches, deduplicated the records, wrote the methodology section, and reviewed the pre-submission manuscript.

## Methodology

An information specialist (EH) retrieved relevant records published in the English language about the perspectives of healthcare professionals and patients on the factors influencing the adoption of diagnostic artificial intelligence in clinical practice by searching the following databases on 22/02/2023 and updated the search results on 08/02/2024: Ovid MEDLINE; PubMed; Ovid Embase; Scopus; CINAHL via EBSCOhost; and the Web of Science – All Databases. Search filters for qualitative studies were used for PubMed and CINAHL from the CADTH Search Filters Database. The search strategies used text words and relevant indexing terms where applicable. The full strategies are available in the appendix. All references were exported to EndNote 20 (Thomson Reuters, New York, NY) and duplicates were removed using the Systematic Review Accelerator Deduplicator tool (https://sr-accelerator.com/#/deduplicator - Thorough Deduplicator Algorithm).

**References**

Qualitative Studies - PubMed. In: CADTH Search Filters Database. Ottawa: CADTH; 2023: [https://searchfilters.cadth.ca/link/93. Accessed 2023-01-27](https://searchfilters.cadth.ca/link/93.%20Accessed%202023-01-27).

Qualitative Studies - CINAHL. In: CADTH Search Filters Database. Ottawa: CADTH; 2023: <https://searchfilters.cadth.ca/link/92>. Accessed 2023-02-22.

## Search Results

|  | Search results 22/02/2023 in full | Search results 08/02/2024 (2022 to search date only) |
| --- | --- | --- |
| Ovid MEDLINE | 5129 | 3036 |
| PubMed | 1690 | 1146 |
| Ovid Embase | 5170 | 3693 |
| Scopus | 9001 | 6056 |
| EBSCOhost CINAHL | 1146 | 469 |
| Web of Science All Databases | 8283 | 6806 |
| Total | 30419 | 21206 |
| Total after deduplication (SR Accelerator method) | 16734 | - |
| Unique publications added to the databases since 22/02/2023 | - | 6665 |

## Search Strategies

**Database: Medline (Ovid MEDLINE® Epub Ahead of Print, In-Process & Other Non-Indexed Citations, Ovid MEDLINE® Daily and Ovid MEDLINE®) 1946 to present**

Link to search history: <https://ovidsp.ovid.com/ovidweb.cgi?T=JS&NEWS=N&PAGE=main&SHAREDSEARCHID=3wWDaU0JU6Zluq67e0MW2fBPuCK0ICpx5iLd7rwfyeprcsj7fJPbW867m8Pc3oua>
**Search Strategy:**
**1**  exp Artificial Intelligence/ (189058)
**2**  ("machine learning" or "deep learning" or "artificial neural network*" or "deep neural network*" or "convolutional neural network*").ti,ab. (164456)
**3**  "artificial intelligence".ti,ab. (38291)
**4**  "machine learning".ti. and deep.ti,ab. (3037)
**5**  (ensemble and deep).ti,ab. (2660)
**6**  ("reinforcement learning" or "deep belief network*" or "recurrent neural network*" or "feedforward neural network*").ti,ab. (11489)
**7**  "feed forward neural network*".ti,ab. (783)
**8**  ("boltzmann machine*" or "long short-term memory" or "gated recurrent unit*" or "rectified linear unit*" or autoencoder or "auto-encoder" or backpropagation or "multilayer perceptron" or "multi-layer perceptron" or convnet or "convolutional learning").ti,ab. (14978)
**9**  1 or 2 or 3 or 4 or 5 or 6 or 7 or 8 (297173)
**10**  "Attitude of Health Personnel"/ (132245)
**11**  exp Health Personnel/ (626278)
**12**  exp Patient Care Team/ (73140)
**13**  (health?care adj2 (profession* or worker* or provider* or staff or personnel)).ti,ab. (105698)
**14**  (staff or "healthcare workforce" or physician* or clinician* or stakeholders or "healthcare professional*").ti,ab. (1007161)
**15**  (doctor* or clinician* or "family practition*" or "general practition*" or nurs* or obstetrician* or physician* or neurologist* or "health profession*" or (health?care adj2 (profession* or worker* or provider* or staff or personnel)) or "nursing staff" or "medical assist*" or "public health practition*" or consultant* or "care practition*" or "medical practition*" or p?ediatric*).tw. (1968234)
**16**  exp Patients/ (85125)
**17**  (radiolog* or adopter*).ti,ab. (319988)
**18**  (patient or patients or participants).ti,ab. (9116437)
**19**  ("the public" or "general public").ti,ab. (614771)
**20**  10 or 11 or 12 or 13 or 14 or 15 or 16 or 17 or 18 or 19 (10833828)
**21**  exp qualitative research/ (85754)
**22**  interview/ (30934)
**23**  focus groups/ or interviews as topic/ or exp "surveys and questionnaires"/ (1304001)
**24**  (qualitative* or survey* or "focus group*" or interview*).tw. (1526211)
**25**  (phenomenological or experienc* or perception* or perceiv* or "grounded theory").tw. (1931890)
**26**  (perspective* or questionnaire*).tw. (1145858)
**27**  ((("semi-structured" or semistructured or unstructured or informal or "in-depth" or indepth or "face-to-face" or structured or guide) adj3 (interview* or discussion* or questionnaire*)) or (focus group* or qualitative or ethnograph* or fieldwork or "field work" or "key informant")).ti,ab. or narration/ (499909)
**28**  21 or 22 or 23 or 24 or 25 or 26 or 27 (4309636)
**29**  "Patient Acceptance of Health Care"/ (55526)
**30**  exp "diffusion of innovation"/ (21637)
**31**  (implement* or adopt* or accept* or "diffusion of innovation" or aware* or uptake or up-take or takeup or take-up or adhere or adhered or adherence or concordance or accordance or comply or complies or compliance or complying or disseminat* or spread or spreading or barrier or barriers or facilitat*).ti,ab. (3890262)
**32**  (diffusion or "knowledge translation").ti,ab. (251780)
**33**  29 or 30 or 31 or 32 (4131528)
**34**  9 and 20 and 28 and 33 (6778)
**35**  34 (6778)
**36**  limit 35 to english language (6654)
**37**  limit 36 to yr="2022 -Current" (3036)

**PubMed**

(((((((((("Artificial Intelligence"[Mesh]) OR ("machine learning"[Title/Abstract] OR "deep learning"[Title/Abstract] OR "artificial neural network*"[Title/Abstract] OR "deep neural network*"[Title/Abstract] OR "convolutional neural network*"[Title/Abstract])) OR ("artificial intelligence"[Title/Abstract])) OR (("machine learning"[Title]) AND (deep[Title/Abstract]))) OR (ensemble[Title/Abstract] AND deep[Title/Abstract])) OR ("reinforcement learning"[Title/Abstract] OR "deep belief network*"[Title/Abstract] OR "recurrent neural network*"[Title/Abstract] OR "feedforward neural network*"[Title/Abstract])) OR ("feed forward neural network*"[Title/Abstract])) OR ("boltzmann machine*"[Title/Abstract] OR "long short-term memory"[Title/Abstract] OR "gated recurrent unit*"[Title/Abstract] OR "rectified linear unit*"[Title/Abstract] OR autoencoder[Title/Abstract] OR "auto-encoder"[Title/Abstract] OR backpropagation[Title/Abstract] OR "multilayer perceptron"[Title/Abstract] OR "multi-layer perceptron"[Title/Abstract] OR convnet[Title/Abstract] OR "convolutional learning"[Title/Abstract])) AND ((((((((("Attitude of Health Personnel"[Mesh]) OR "Health Personnel"[Mesh]) OR "Patient Care Team"[Mesh]) OR (("health care"[Title/Abstract] OR healthcare[Title/Abstract]) AND (profession*[Title/Abstract] OR worker*[Title/Abstract] OR provider*[Title/Abstract] OR staff[Title/Abstract] OR personnel[Title/Abstract]))) OR (staff[Title/Abstract] OR "healthcare workforce"[Title/Abstract] OR physician*[Title/Abstract] OR clinician*[Title/Abstract] OR stakeholders[Title/Abstract] OR "healthcare professional*"[Title/Abstract])) OR (doctor*[Title/Abstract] OR "family practition*"[Title/Abstract] OR "general practition*"[Title/Abstract] OR nurs*[Title/Abstract] OR obstetrician*[Title/Abstract] OR neurologist*[Title/Abstract] OR "health profession*"[Title/Abstract] OR (health?care adj2 (profession*[Title/Abstract] OR worker*[Title/Abstract] OR provider*[Title/Abstract] OR staff[Title/Abstract] OR personnel[Title/Abstract])) OR "nursing staff"[Title/Abstract] OR "medical assist*"[Title/Abstract] OR "public health practition*"[Title/Abstract] OR consultant*[Title/Abstract] OR "care practition*"[Title/Abstract] OR "medical practition*"[Title/Abstract] OR p?ediatric*[Title/Abstract])) OR ("Patients"[Mesh])) OR ((radiolog*[Title/Abstract] OR adopter*[Title/Abstract]) AND (patient[Title/Abstract] OR patients[Title/Abstract] OR participants[Title/Abstract]))) OR ("the public"[Title/Abstract] OR "general public"[Title/Abstract]))) AND ("Empirical Research"[mh] OR "Interview"[pt] OR "Interviews as Topic"[mh] OR "Personal Narrative"[pt] OR "Personal Narratives as Topic"[mh] OR "Focus Groups"[mh] OR "Narration"[mh] OR "Nursing Methodology Research"[mh] OR interview*[tiab] OR theme[tiab] OR thematic[tiab] OR "qualitative"[all fields] OR questionnaire*[all fields] OR "ethnological research"[all fields] OR ethnograph*[all fields] OR ethnonursing[all fields] OR phenomenol*[all fields] OR "grounded theor*"[all fields] OR "grounded stud*"[all fields] OR "grounded research"[all fields] OR "grounded analys*"[all fields] OR "life stor*"[all fields] OR "women's stor*"[all fields] OR emic[all fields] OR etic[all fields] OR hermeneutic*[all fields] OR heuristic*[all fields] OR "semiotic"[all fields] OR "data saturat*"[tiab] OR "participant observ*"[tiab] OR "social construct*"[tiab] OR postmodern*[tiab] OR "post-structural*"[tiab] OR "post structural*"[tiab] OR poststructural*[tiab] OR "post modern*"[tiab] OR "post-modern*"[tiab] OR feminis*[tiab] OR action research[tiab] OR "cooperative inquir*"[tiab] OR "co-operative inquir*"[tiab] OR humanistic[tiab] OR existential[tiab] OR experiential[tiab] OR paradigm*[tiab] OR "field stud*"[tiab] OR "field research"[tiab] OR "human science"[tiab] OR "biographical method*"[tiab] OR "theoretical sampl*"[all fields] OR "purposeful sampl*"[tiab] OR "purposive sampl*"[tiab] OR "focus group*"[all fields] OR account[tiab] OR accounts[tiab] OR unstructured[tiab] OR "open-ended"[tiab] OR texts[tiab] OR textual[tiab] OR narrative*[tiab] OR narration[tiab] OR "life world"[tiab] OR "life-world"[tiab] OR "conversation analys*"[tiab] OR "personal experience*"[tiab] OR "theoretical saturation"[tiab] OR "lived experience*"[tiab] OR "life experience*"[tiab] OR "cluster sampl*"[tiab] OR "observational method*"[tiab] OR "content analysis"[tiab] OR "constant comparative"[tiab] OR "constant comparison"[tiab] OR "discourse analys*"[tiab] OR "discursive analys*"[tiab] OR "narrative analys*"[tiab] OR Heidegger*[tiab] OR colaizzi*[tiab] OR spiegelberg*[tiab] OR "van manen*"[tiab] OR "van kaam*"[tiab] OR "merleau ponty*"[tiab] OR Husserl*[tiab] OR Foucault[tiab] OR ricoeur[tiab] OR glaser*[tiab] OR (corbin*[tiab] AND strauss*[tiab]))) AND ((("Patient Acceptance of Health Care"[Mesh]) OR "Diffusion of Innovation"[Mesh]) OR (implement*[Title/Abstract] OR adopt*[Title/Abstract] OR accept*[Title/Abstract] OR "diffusion of innovation"[Title/Abstract] OR aware*[Title/Abstract] OR uptake[Title/Abstract] OR up-take[Title/Abstract] OR takeup[Title/Abstract] OR take-up[Title/Abstract] OR adhere[Title/Abstract] OR adhered[Title/Abstract] OR adherence[Title/Abstract] OR concordance[Title/Abstract] OR accordance[Title/Abstract] OR comply[Title/Abstract] OR complies[Title/Abstract] OR compliance[Title/Abstract] OR complying[Title/Abstract] OR disseminat*[Title/Abstract] OR spread[Title/Abstract] OR spreading[Title/Abstract] OR barrier[Title/Abstract] OR barriers[Title/Abstract] OR facilitat*[Title/Abstract] OR diffusion[Title/Abstract] OR "knowledge translation"[Title/Abstract])) Filters: **English, from 2022 - 2024**

**Database: Embase 1974 to present**

Link to search history: <https://ovidsp.ovid.com/ovidweb.cgi?T=JS&NEWS=N&PAGE=main&SHAREDSEARCHID=5fO8OaDs12NaFv92oGU7Tm1jjZFRo6r0FRXeRV4BRPmPLbGn8YpfW6E52g8MIQUAZ>
**Search Strategy:**
**1**  exp artificial intelligence/ (95816)
**2**  ("machine learning" or "deep learning" or "artificial neural network*" or "deep neural network*" or "convolutional neural network*").ti,ab. (193353)
**3**  "artificial intelligence".ti,ab. (45932)
**4**  "machine learning".ti. and deep.ti,ab. (3712)
**5**  (ensemble and deep).ti,ab. (2899)
**6**  ("reinforcement learning" or "deep belief network*" or "recurrent neural network*" or "feedforward neural network*").ti,ab. (12823)
**7**  "feed forward neural network*".ti,ab. (1005)
**8**  ("boltzmann machine*" or "long short-term memory" or "gated recurrent unit*" or "rectified linear unit*" or autoencoder or "auto-encoder" or backpropagation or "multilayer perceptron" or "multi-layer perceptron" or convnet or "convolutional learning").ti,ab. (16162)
**9**  1 or 2 or 3 or 4 or 5 or 6 or 7 or 8 (282463)
**10**  health personnel attitude/ (88832)
**11**  exp health care personnel/ (2031272)
**12**  (health?care adj2 (profession* or worker* or provider* or staff or personnel)).ti,ab. (144516)
**13**  (staff or "healthcare workforce" or physician* or clinician* or stakeholders or "healthcare professional*").ti,ab. (1425758)
**14**  (doctor* or clinician* or "family practition*" or "general practition*" or nurs* or obstetrician* or physician* or neurologist* or "health profession*" or (health?care adj2 (profession* or worker* or provider* or staff or personnel)) or "nursing staff" or "medical assist*" or "public health practition*" or consultant* or "care practition*" or "medical practition*" or p?ediatric*).tw. (2705429)
**15**  exp patient/ (3057148)
**16**  (radiolog* or adopter*).ti,ab. (464408)
**17**  (patient or patients or participants).ti,ab. (13188485)
**18**  ("the public" or "general public").ti,ab. (735454)
**19**  10 or 11 or 12 or 13 or 14 or 15 or 16 or 17 or 18 (15591695)
**20**  exp qualitative research/ (124200)
**21**  exp interview/ (382292)
**22**  exp questionnaire/ (956630)
**23**  (qualitative* or survey* or "focus group*" or interview*).tw. (1922980)
**24**  (phenomenological or experienc* or perception* or perceiv* or "grounded theory").tw. (2601549)
**25**  (perspective* or questionnaire*).tw. (1524771)
**26**  ((("semi-structured" or semistructured or unstructured or informal or "in-depth" or indepth or "face-to-face" or structured or guide) adj3 (interview* or discussion* or questionnaire*)) or (focus group* or qualitative or ethnograph* or fieldwork or "field work" or "key informant")).ti,ab. (612453)
**27**  ethnography/ (3952)
**28**  20 or 21 or 22 or 23 or 24 or 25 or 26 or 27 (5061036)
**29**  "diffusion of innovation"/ (187)
**30**  (implement* or adopt* or accept* or "diffusion of innovation" or aware* or uptake or up-take or takeup or take-up or adhere or adhered or adherence or concordance or accordance or comply or complies or compliance or complying or disseminat* or spread or spreading or barrier or barriers or facilitat*).ti,ab. (5006260)
**31**  (diffusion or "knowledge translation").ti,ab. (296768)
**32**  29 or 30 or 31 (5239432)
**33**  9 and 19 and 28 and 32 (7046)
**34**  limit 33 to english language (6930)
**35**  limit 34 to yr="2022 -Current" (3693)

**SCOPUS**

( TITLE-ABS-KEY ( ( "machine learning" OR "deep learning" OR "artificial neural network*" OR "deep neural network*" OR "convolutional neural network*" OR "artificial intelligence" OR ( ensemble AND deep ) OR "reinforcement learning" OR "deep belief network*" OR "recurrent neural network*" OR "feedforward neural network*" OR "feed forward neural network*" OR "boltzmann machine*" OR "long short-term memory" OR "gated recurrent unit*" OR "rectified linear unit*" OR autoencoder OR "auto-encoder" OR backpropagation OR "multilayer perceptron" OR "multi-layer perceptron" OR convnet OR "convolutional learning" ) ) ) AND ( ( TITLE-ABS-KEY ( qualitative* OR survey* OR "focus group*" OR interview* OR phenomenological OR experienc* OR perception* OR perceiv* OR "grounded theory" OR perspective* OR questionnaire* ) OR TITLE-ABS-KEY ( ( ( ( "semi-structured" OR semistructured OR unstructured OR informal OR "in-depth" OR indepth OR "face-to-face" OR structured OR guide ) W/3 ( interview* OR discussion* OR questionnaire* ) ) OR ( "focus group*" OR ethnograph* OR fieldwork OR "field work" OR "key informant" ) ) ) ) ) AND ( TITLE-ABS-KEY ( implement* OR adopt* OR accept* OR "diffusion of innovation" OR aware* OR uptake OR up-take OR takeup OR take-up OR adhere OR adhered OR adherence OR concordance OR accordance OR comply OR complies OR compliance OR complying OR disseminat* OR spread OR spreading OR barrier OR barriers OR facilitat* OR ( diffusion W/6 innovation ) OR "knowledge translation" ) ) AND ( ( TITLE-ABS-KEY ( health* W/2 ( profession* OR worker* OR provider* OR staff OR personnel ) ) ) OR ( TITLE-ABS-KEY ( staff OR "healthcare workforce" OR physician* OR clinician* OR stakeholders OR "healthcare professional*" OR doctor* OR "family practition*" OR "general practition*" OR nurs* OR obstetrician* OR neurologist* OR "health profession*" ) ) OR ( TITLE-ABS-KEY ( "nursing staff" OR "medical assist*" OR "public health practition*" OR consultant* OR "care practition*" OR "medical practition*" OR pediatric* OR paediatric* ) ) OR ( TITLE-ABS-KEY ( radiolog* OR adopter* OR patient OR patients OR participants OR "the public" OR "general public" ) ) ) AND ( LIMIT-TO ( LANGUAGE , "English" ) ) 2022-2024

Top of Form

| 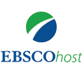CINAHL | Wednesday, February 22, 2023 2:39:58 PM |
| --- | --- |

Bottom of Form

Top of Form

| **#** | **Query** | **Results** |
| --- | --- | --- |
| S1 | (MH "Artificial Intelligence+") | 32,987 |
| S2 | TI ( ("machine learning" or "deep learning" or "artificial neural network*" or "deep neural network*" or "convolutional neural network*") ) OR AB ( ("machine learning" or "deep learning" or "artificial neural network*" or "deep neural network*" or "convolutional neural network*") ) | 0 |
| S3 | TI "artificial intelligence" OR AB "artificial intelligence" | 9,864 |
| S4 | TI ( ensemble and deep ) OR AB ( ensemble and deep ) | 203 |
| S5 | TI ( ("reinforcement learning" or "deep belief network*" or "recurrent neural network*" or "feedforward neural network*") ) OR AB ( ("reinforcement learning" or "deep belief network*" or "recurrent neural network*" or "feedforward neural network*") ) | 722 |
| S6 | TI "feed forward neural network*" OR AB "feed forward neural network*" | 45 |
| S7 | TI ( ("boltzmann machine*" or "long short-term memory" or "gated recurrent unit*" or "rectified linear unit*" or autoencoder or "auto-encoder" or backpropagation or "multilayer perceptron" or "multi-layer perceptron" or convnet or "convolutional learning") ) OR AB ( ("boltzmann machine*" or "long short-term memory" or "gated recurrent unit*" or "rectified linear unit*" or autoencoder or "auto-encoder" or backpropagation or "multilayer perceptron" or "multi-layer perceptron" or convnet or "convolutional learning") ) | 888 |
| S8 | S1 OR S2 OR S3 OR S4 OR S5 OR S6 OR S7 | 49,932 |
| S9 | (MH "Attitude of Health Personnel") | 55,476 |
| S10 | (MH "Health Personnel+") | 641,471 |
| S11 | TI ( health* n2 (profession* or worker* or provider* or staff or personnel) ) OR AB ( health* n2 (profession* or worker* or provider* or staff or personnel) ) | 201,965 |
| S12 | TI ( staff or "healthcare workforce" or physician* or clinician* or stakeholders or "healthcare professional*" or doctor* or "family practition*" or "general practition*" or nurs* or obstetrician* or neurologist* or "health profession*" or "nursing staff" or "medical assist*" or "public health practition*" or consultant* or "care practition*" or "medical practition*" or paediatric* OR pediatric* ) OR AB ( staff or "healthcare workforce" or physician* or clinician* or stakeholders or "healthcare professional*" or doctor* or "family practition*" or "general practition*" or nurs* or obstetrician* or neurologist* or "health profession*" or "nursing staff" or "medical assist*" or "public health practition*" or consultant* or "care practition*" or "medical practition*" or paediatric* OR pediatric* ) | 1,227,703 |
| S13 | (MH "Patients+") | 329,101 |
| S14 | TI ( radiolog* or adopter or patient or patients or participants or "the public" or "general public" ) OR AB ( radiolog* or adopter or patient or patients or participants or "the public" or "general public" ) | 2,716,370 |
| S15 | S9 OR S10 OR S11 OR S12 OR S13 OR S14 | 3,782,792 |
| S16 | MH Qualitative Studies OR MH Grounded theory OR MH Narratives OR MH Interviews+ OR MH Audiorecording OR MH Focus Groups OR MH Research, Nursing OR MH Discourse Analysis OR MH Content Analysis OR MH Ethnographic Research OR MH Ethnonursing Research OR MH Constant Comparative Method OR MH Qualitative Validity+ OR MH Purposive Sample OR MH Observational Methods+ OR MH Field Studies OR MH Theoretical Sample OR MH Phenomenology OR MH Phenomenological Research OR MH Life Experiences+ OR MH Cluster Sample+ OR TI qualitative OR AB qualitative OR TI interview* OR AB interview* OR TI (theme* or thematic) OR AB (theme* or thematic) OR TI ("ethnological research") OR AB ("ethnological research") OR TI ethnonursing OR AB ethnonursing OR TI ethnograph* OR AB ethnograph* OR TI phenomenol* OR AB phenomenol* OR TI "focus group*" OR AB "focus group*" OR TI (grounded N1 (theor* OR analys?s OR research OR studies OR study)) OR AB (grounded N1 (theor* OR analys?s OR research OR studies OR study)) OR TI ("life stor*") OR AB ("life stor*") OR TI (emic OR etic OR hermeneutic* OR heuristic* OR semiotic) OR AB (emic OR etic OR hermeneutic* OR heuristic* OR semiotic) OR TI (data N1 saturat*) OR AB (data N1 saturat*) OR TI ("participant observ*") OR AB ("participant observ*") OR TI ("social construct*" OR postmodern* OR "post-structural*" OR poststructural* OR "post-modern*" OR feminis*) OR AB ("social construct*" OR postmodern* OR "post-structural*" OR poststructural* OR "post-modern*" OR feminis*) OR TI ("action research" OR "cooperative inquir*" OR "co-operative inquir*") OR AB ("action research" OR "cooperative inquir*" OR "co-operative inquir*") OR TI (humanistic OR existential OR experiential OR paradigm*) OR AB (humanistic OR existential OR experiential OR paradigm*) OR TI (field N1 (research OR study OR studies)) OR AB (field N1 (research OR study OR studies)) OR TI "human science" OR AB "human science" OR TI "biographical method" OR AB "biographical method" OR TI ("theoretical sampl*") OR AB ("theoretical sampl*") OR TI ("purpos* N4 sampl*") OR AB ("purpos* N4 sampl*") OR TI ("open-ended" OR narrative* OR textual OR texts OR "semi-structured") OR AB ("open-ended" OR narrative* OR textual OR texts OR "semi-structured") OR TI ("life world" OR "life-world" OR "conversation analys?s" OR "personal experience*" OR "theoretical saturation") OR AB ("life world" OR "life-world" OR "conversation analys?s" OR "personal experience*" OR "theoretical saturation") OR TI ((life OR lived) N1 experience*) OR AB ((life OR lived) N1 experience*) OR TI ("cluster sampl*") OR AB ("cluster sampl*") OR TI ("observational method*") OR AB ("observational method*") OR TI ("content analysis") OR AB ("content analysis") OR TI ((discurs* OR discourse*) N3 analys?s) OR AB ((discurs* OR discourse*) N3 analys?s) OR TI (constant N1 (comparison OR comparative)) OR AB (constant N1 (comparison OR comparative)) OR TI ("narrative analys?s") OR AB ("narrative analys?s") OR TI (heidegger* OR colaizzi* OR spiegelberg* OR merleau* OR husserl* OR foucault* OR ricoeur OR glaser*) OR AB (heidegger* OR colaizzi* OR spiegelberg* OR merleau* OR husserl* OR foucault* OR ricoeur OR glaser*) OR TI (van N1 manen*) OR AB (van N1 manen*) OR TI (van N1 kaam*) OR AB (van N1 kaam*) OR TI (Corbin* N2 strauss*) OR AB (Corbin* N2 strauss*) | 757,491 |
| S17 | (MH "Diffusion of Innovation+") | 20,291 |
| S18 | TI ( implement* or adopt* or accept* or "diffusion of innovation" or aware* or uptake or up-take or takeup or take-up or adhere or adhered or adherence or concordance or accordance or comply or complies or compliance or complying or disseminat* or spread or spreading or barrier or barriers or facilitat* ) OR AB ( implement* or adopt* or accept* or "diffusion of innovation" or aware* or uptake or up-take or takeup or take-up or adhere or adhered or adherence or concordance or accordance or comply or complies or compliance or complying or disseminat* or spread or spreading or barrier or barriers or facilitat* ) | 932,439 |
| S19 | TI ( diffusion or "knowledge translation" ) OR AB ( diffusion or "knowledge translation" ) | 25,936 |
| S20 | S17 OR S18 OR S19 | 965,066 |
| S21 | S8 AND S15 AND S16 AND S20 | 1,359 |
| S22 | S8 AND S15 AND S16 AND S20 | 476 |
| S23 | S8 AND S15 AND S16 AND S20 | 469 |

Bottom of Form

**Web of Science All Databases**

"machine learning" OR "deep learning" OR "artificial neural network*" OR "deep neural network*" OR "convolutional neural network*" OR "artificial intelligence" OR ( ensemble AND deep ) OR "reinforcement learning" OR "deep belief network*" OR "recurrent neural network*" OR "feedforward neural network*" OR "feed forward neural network*" OR "boltzmann machine*" OR "long short-term memory" OR "gated recurrent unit*" OR "rectified linear unit*" OR autoencoder OR "auto-encoder" OR backpropagation OR "multilayer perceptron" OR "multi-layer perceptron" OR convnet OR "convolutional learning" (Topic) and qualitative* OR survey* OR "focus group*" OR interview* OR phenomenological OR experienc* OR perception* OR perceiv* OR "grounded theory" OR perspective* OR questionnaire* or "focus group*" OR ethnograph* OR fieldwork OR "field work" OR "key informant" (Topic) and implement* OR adopt* OR accept* OR "diffusion of innovation" OR aware* OR uptake OR up-take OR takeup OR take-up OR adhere OR adhered OR adherence OR concordance OR accordance OR comply OR complies OR compliance OR complying OR disseminat* OR spread OR spreading OR barrier OR barriers OR facilitat* OR diffusion OR "knowledge translation" (Topic) and (health* near/2 (profession* or worker* or provider* or staff or personnel)) OR "healthcare workforce" OR physician* OR clinician* OR stakeholders OR "healthcare professional*" OR doctor* OR "family practition*" OR "general practition*" OR nurs* OR obstetrician* OR neurologist* OR "health profession*" OR "nursing staff" OR "medical assist*" OR "public health practition*" OR consultant* OR "care practition*" OR "medical practition*" OR pediatric* OR paediatric* OR radiolog* OR adopter* OR patient OR patients OR participants OR "the public" OR "general public" (Topic) and Preprint Citation Index (Exclude – Database) and English (Languages) 2002-4
