# Supplementary material for: Stakeholder perspectives towards diagnostic artificial intelligence: a co-produced qualitative evidence synthesis
Source: eClinicalMedicine. 2024 Mar 22;71:102555. doi: 10.1016/j.eclinm.2024.102555 (PMC10973718; doi:10.1016/j.eclinm.2024.102555)
Supplement: Supplementary Material 4 [file mmc2.docx]

| **Title** | **Year** | **Journal** | **Authors** | **Title/abstract vote 1** | **Title/abstract vote 2** | **Full text vote 1** | **Full text vote 2** | **Exclusion reason 1** | **Exclusion reason 2** | **Exclusion reason in Figure 1** |
| --- | --- | --- | --- | --- | --- | --- | --- | --- | --- | --- |
| Assessment of the willingness of radiologists and radiographers to accept the integration of artificial intelligence into radiology practice | 2022 | Academic radiology | Abuzaid et al. | Y - RK | Y - AF | N - RK | N - AF | Not primary qualitative research | Not primary qualitative research | Not primary qualitative research |
| An extensive survey of radiographers from the Middle East and India on artificial intelligence integration in radiology practice | 2021 | Health and technology | Abuzaid et al. | Y - RK | Y - AF | N - RK | N - AF | Not primary qualitative research | Not primary qualitative research | Not primary qualitative research |
| Integration of artificial intelligence into nursing practice | 2022 | Health and technology | Abuzaid et al. | Y - RK | Maybe - JC | N - RK | N - JS | Not primary qualitative research | Not primary qualitative research | Not primary qualitative research |
| Exploring patient perspectives on how they can and should be engaged in the development of artificial intelligence (AI) applications in healthcare | 2023 | BMC health services research | Adus et al. | Maybe – RK | Maybe – AF | Maybe – RK | Maybe - AF | Diagnostic AI not explicitly investigated | Diagnostic AI not explicitly investigated | Diagnostic AI not explicitly investigated |
| Patient perceptions on data sharing and applying artificial intelligence to health care data | 2021 | Journal of medical internet research | Aggarwal et al. | Maybe - JC | Y - RK | N - RK | N - AF | Not primary qualitative research | Not primary qualitative research | Not primary qualitative research |
| Knowledge, attitude, and practice of artificial intelligence among doctors and medical students in Pakistan: a cross-sectional online survey | 2022 | Annals of medicine and surgery | Ahmed et al. | N - RK | Maybe - JS | N - RK | N - DJ | Not primary qualitative research | Not primary qualitative research | Not primary qualitative research |
| The knowledge and attitudes of the public toward the clinical use of artificial intelligence | 2021 | Asian journal of pharmaceutics | Ahmed et al. | Y - JS | Y - RK | N - RK | N - SD | Not primary qualitative research | Not primary qualitative research | Not primary qualitative research |
| Knowledge, perceptions, and expectations of artificial intelligence in radiography practice: a global radiography workforce survey | 2022 | Journal of medical imaging and radiation sciences | Akudjedu et al. | Maybe - AF | Y - RK | N - RK | N - JC | Not primary qualitative research | Not primary qualitative research | Not primary qualitative research |
| Artificial intelligence in patient care in Riyadh, Saudi Arabia 2019-2020 | 2021 | Medical science | Al Fadeel et al. | Maybe - JC | Y - RK | N - RK | N - AF | Not primary qualitative research | Not primary qualitative research | Not primary qualitative research |
| Evaluation of physician perspectives of artificial intelligence in ophthalmology: a pilot study | 2020 | Investigative ophthalmology and visual science | Al-Khaled et al. | Y - AF | Y - RK | N - RK | N - JS | Conference abstract | Conference abstract | Conference abstract |
| Examining the effect of explanation on satisfaction and trust in AI diagnostic systems | 2021 | BMC Medical Informatics and Decision Making | Alam and Mueller | Maybe - RK | N - JS | N - RK | N - AF | Not primary qualitative research | Not primary qualitative research | Not primary qualitative research |
| Artificial intelligence and the future of anesthetiology: qualitative findings from a national survey of physician anesthesiologists | 2022 | Anesthesia and Analgesia | Alamo et al. | Maybe - RK | Y - AF | N - RK | N - AF | Conference abstract | Conference abstract | Conference abstract |
| Acceptance of artificial intelligence (AI) and machine learning (ML) among radiologists in Saudi Arabia | 2022 | International journal of advanced and applied sciences | Alamoudi | Y - RK | Y - AF | N - RK | N - JC | Not primary qualitative research | Not primary qualitative research | Not primary qualitative research |
| Clinicians’ views on using artificial intelligence in healthcare: opportunities, challenges, and beyond | 2023 | Cureus | Alanazi | Maybe – RK | Maybe – AF | N – AF | N - RK | Diagnostic AI not explicitly investigated | Diagnostic AI not explicitly investigated | Diagnostic AI not explicitly investigated |
| Perspectives of radiographers on the emergence of artificial intelligence in diagnostic imaging in Saudi Arabia | 2022 | Insights into imaging | Aldhafeeri | Y - RK | Y - AF | N - RK | N - JS | Not primary qualitative research | Not primary qualitative research | Not primary qualitative research |
| Radiology community attitude in Saudi Arabia about the applications of artificial intelligence in radiology | 2021 | Healthcare | Alelyani et al. | Y - RK | Y - AF | N - RK | N - AF | Not primary qualitative research | Not primary qualitative research | Not primary qualitative research |
| Perspective toward machine learning implementation in pediatric medicine: mixed methods study | 2022 | JMIR medical informatics | Alexander et al. | Y - AF | Y - RK | N - RK | N - AF | Diagnostic AI not explicitly investigated | Diagnostic AI not explicitly investigated | Diagnostic AI not explicitly investigated |
| Medical students’ attitudes and perceptions toward artificial intelligence applications | 2022 | Journal for educators, teachers and trainers | Ali et al. | Y - RK | Y - AF | N - RK | N - AF | Not primary qualitative research | Not primary qualitative research | Not primary qualitative research |
| Machine learning in otorhinolaryngology, head and neck surgery and its applications in diagnosis and management: undergraduates perception toward new era | 2021 | World family medicine | Alomary et al. | Y - RK | Y - JS | N - RK | N - SD | Not primary qualitative research | Not primary qualitative research | Not primary qualitative research |
| Navigating the doctor-patient-AI relationship – a mixed-methods study of physician attitudes toward artificial intelligence in primary care | 2024 | BMC primary care | Allen et al. | Maybe – RK | Maybe – AF | N – AF | N - RK | Diagnostic AI not explicitly investigated | Diagnostic AI not explicitly investigated | Diagnostic AI not explicitly investigated |
| 2020 ACR Data Science Institute artificial intelligence survey | 2021 | Journal Am Coll Radiol | Allen et al. | N - RK | Y - AF | N - RK | N - AF | Not primary qualitative research | Not primary qualitative research | Not primary qualitative research |
| Are physicians and medical students ready for artificial intelligence applications in healthcare? | 2023 | Digital health | AlZaabi | Y - RK | N - AF | N - RK | N - AF | Not primary qualitative research | Not primary qualitative research | Not primary qualitative research |
| Artificial intelligence and the medical physics profession – a Swedish perspective | 2021 | Phys Med | Andersson et al. | Y - RK | N - AF | N - RK | N - DJ | Not primary qualitative research | Not primary qualitative research | Not primary qualitative research |
| Technology for early detection of depression and anxiety in older people | 2017 | Studies in health technology and informatics | Andrews et al. | Maybe - JS | N - RK | N - RK | N - AF | Diagnostic AI not explicitly investigated | Diagnostic AI not explicitly investigated | Diagnostic AI not explicitly investigated |
| Artificial intelligence in medical imaging practice in Africa: a qualitative content analysis study of radiographers’ perspectives | 2021 | Insights into imaging | Antwi et al. | Maybe - JS | Maybe - RK | N - RK | N - JC | Not primary qualitative research | Not primary qualitative research | Not primary qualitative research |
| Integrating ethics in AI development: a qualitative study | 2024 | BMC Med Ethics | Arbelaez Ossa et al. | Maybe – AF | Maybe – RK | N – AF | N - RK | Diagnostic AI not explicitly investigated | Diagnostic AI not explicitly investigated | Diagnostic AI not explicitly investigated |
| Clinical laboratory employees’ attitudes toward artificial intelligence | 2020 | Laboratory medicine | Ardon and Schmidt | Maybe - RK | Y - AF | N - RK | N - AF | Not primary qualitative research | Not primary qualitative research | Not primary qualitative research |
| A survey of pregnant patients’ perspectives on the implementation of artificial intelligence in clinical care | 2022 | Journal of the American medical informatics association: JAMIA | Armero et al. | Y - RK | Y - JS | N - RK | N - AF | Not primary qualitative research | Not primary qualitative research | Not primary qualitative research |
| Explainability for artificial intelligence in healthcare: a multidisciplinary perspective | 2020 | BMC medical informatics and decision making | Amann et al. | N – RK | Y - RH | N - RK | N - SD | Commentary/editorial | Systematic review | Commentary/editorial |
| AI diagnostic technologies and the gap in colorectal cancer screening participation | 2022 | Studies in health technology and informatics | Ameen et al. | Maybe - RK | Maybe - JS | N - RK | N - DJ | Conference abstract | Conference abstract | Conference abstract |
| The effects of integrated IT support on the prehospital stroke process: results from a realistic experiment | 2019 | Journal of healthcare informatics research | Andersson et al. | Maybe - JS | Y - RK | N - RK | N - AF | Diagnostic AI not explicitly investigated | Diagnostic AI not explicitly investigated | Diagnostic AI not explicitly investigated |
| Exploring perceptions of healthcare technologies enabled by artificial intelligence: an online, scenario-based study | 2021 | BMC medical informatics and decision making | Antes et al. | Y - RK | Y - DJ | N - RK | N - JS | Not primary qualitative research | Not primary qualitative research | Not primary qualitative research |
| A cross-sectional survey on an insight into the current perceptions of Indian radiologists, radiographers, radiology trainees and medical imaging students on the future impact of artificial intelligence (AI) on the profession | 2023 | Journal of pharmaceutical negative results | Ansari et al. | Y - AF | Y - RK | N - RK | N - AF | Not primary qualitative research | Not primary qualitative research | Not primary qualitative research |
| Artificial intelligence (AI) healthcare technology innovations: the current state and challenges from a life science industry perspective | 2021 | Technology analysis and strategic management | Apell and Eriksson | Y - RK | Y - AF | N - RK | N - AF | Diagnostic AI not explicitly investigated | Diagnostic AI not explicitly investigated | Diagnostic AI not explicitly investigated |
| Identifying best practices for clinical decision support and knowledge management in the field | 2010 | Stud Health Technol Inform | Ash et al. | N - JS | Maybe -RK | N - RK | N - AF | Diagnostic AI not explicitly investigated | Diagnostic AI not explicitly investigated | Diagnostic AI not explicitly investigated |
| Interventional radiology and artificial intelligence in radiology: is it time to enhance the vision of our medical students? | 2020 | Insights into Imaging | Auloge et al. | Y - AF | Y - RK | N - RK | N - AF | Not primary qualitative research | Not primary qualitative research | Not primary qualitative research |
| Trust Me, I’m a Doctor-user perceptions of AI-diagnosis for mobile health diagnosis | 2020 | Proceedings of the 19^th^ International Conference on Mobile and Ubiquitous Media | Baldauf et al. | Y - RK | Y - RH | N - RK | N - SD | Conference abstract | Conference abstract | Conference abstract |
| Impact of the rise of artificial intelligence in radiology: what do students think? | 2023 | International journal of environment research and public health | Barreiro-Ares et al. | Maybe - JS | Y - RK | N - RK | N - JC | Not primary qualitative research | Not primary qualitative research | Not primary qualitative research |
| Using artificial intelligence to promote equitable care for inpatients with language barriers and complex medical needs: clinical stakeholder perspectives | 2023 | Journal of the American medical informatics association | Barwise et al. | Maybe – RK | Maybe – AF | N – AF | N - RK | Diagnostic AI not explicitly investigated | Diagnostic AI not explicitly investigated | Diagnostic AI not explicitly investigated |
| Barriers of artificial intelligence in the health sector | 2023 | Intelligent systems reference library | Beltempo et al. | N - RK | Y - AF | N - RK | N - AF | Commentary/editorial | Systematic review | Commentary/editorial |
| Artificial intelligence (AI) acceptance in primary care during the coronavirus pandemic: what is the role of patients’ gender, age and health awareness? A two-phase pilot study | 2023 | Frontiers in public health | Ben-Gal | Maybe - RK | Y - RH | N - RK | N - JS | Not primary qualitative research | Not primary qualitative research | Not primary qualitative research |
| Transparency of artificial intelligence in healthcare: insights from professionals in computing and healthcare worldwide | 2022 | Applied sciences | Bernal and Mazo | Y - RK | Y – AF | N - RK | N - AF | Not primary qualitative research | Not primary qualitative research | Not primary qualitative research |
| Systematic AI support for decision-making in the healthcare sector: obstacles and success factors | 2023 | Health policy and technology | Bertl et al. | Maybe – RK | Maybe – AF | N - RK | N - AF | Not primary qualitative research | Not primary qualitative research | Not primary qualitative research |
| Artificial intelligence and the future of primary care: exploratory qualitative study of UK General Practitioners’ views | 2019 | Journal of medical internet research | Blease et al. | Y - RK | Y - DJ | N - RK | N - AF | Diagnostic AI not explicitly investigated | Diagnostic AI not explicitly investigated | Diagnostic AI not explicitly investigated |
| Research on the clinical translation of health care machine learning: ethicists experiences on lessons learned | 2022 | American Journal of Bioethics | Blumenthal-Barby et al. | Y - RK | Y - JC | N - RK | N - JS | Not primary qualitative research | Not primary qualitative research | Not primary qualitative research |
| Readiness to embrace artificial intelligence among medical doctors and students: a questionnaire-based study | 2022 | JMIR medical education | Boillat et al. | Y - RK | Maybe - RH | N - RK | N - AF | Not primary qualitative research | Not primary qualitative research | Not primary qualitative research |
| A qualitative thematic analysis of addressing the Why: an artificial intelligence (AI) in healthcare symposium | 2022 | Cureus | Borgstadt et al. | Y - RK | Y - AF | N - RK | Y - AF | Diagnostic AI not explicitly investigated |  | Diagnostic AI not explicitly investigated |
| Patient perspectives on artificial intelligence in radiology | 2023 | Journal of the American College of Radiology | Borondy Kitts. | Y – RK | Y – AF | N – RK | N – AF | Not primary qualitative research | Commentary/editorial | Commentary/editorial |
| The integration of artificial intelligence in medical imaging practice: perspectives of African radiographers | 2021 | Radiography | Botwe et al. | Y - RK | Y - AF | N - RK | N - DJ | Not primary qualitative research | Not primary qualitative research | Not primary qualitative research |
| Radiographers’ perspectives on the emerging integration of artificial intelligence into diagnostic imaging: the Ghana study | 2021 | Journal of medical radiation sciences | Botwe et al. | Y - RK | Y - DJ | N - RK | N - AF | Not primary qualitative research | Not primary qualitative research | Not primary qualitative research |
| Improved patient satisfaction and diagnostic accuracy in skin diseases with a visual clinical decision support system- a feasibility study with general practitioners | 2020 | PloS one | Breitbart et al. | Y - RH | Y - RK | N - RK | N - JS | Not primary qualitative research | Not primary qualitative research | Not primary qualitative research |
| BreastScreening-AI: Evaluating medical intelligent agents for human-AI interactions | 2022 | Artificial intelligence in medicine | Calisto et al. | Maybe - RK | Y - DH | N - AF | N - RK | Not primary qualitative research | Not primary qualitative research | Not primary qualitative research |
| Medical students’ perceptions of the impact of artificial intelligence in radiology | 2021 | Percepciones de estudiantes de Medicina sobre el impacto de la intellligencia artificial en radiologia | Caparros and Sendra | N - JC | Y - RK | N - AF | N - RK | Not primary qualitative research | Not primary qualitative research | Not primary qualitative research |
| Perceptions of artificial intelligence among healthcare staff: a qualitative survey study | 2020 | Frontiers in artificial intelligence | Castagno and Khalifa | Y - AF | Y - RK | N - AF | N - RK | Not primary qualitative research | Not primary qualitative research | Not primary qualitative research |
| Doctor resistance of artificial intelligence in healthcare | 2022 | International journal of healthcare information systems and informatics | Chaibi and Zaiem | Maybe – RK | Maybe – AF | N – AF | N - RK | Diagnostic AI not explicitly investigated | Diagnostic AI not explicitly investigated | Diagnostic AI not explicitly investigated |
| Acceptance of clinical artificial intelligence among physicians and medical students: a systematic review with cross-sectional survey | 2022 | Frontiers in Medicine | Chen et al. | Maybe - JC | Maybe - RK | N - DJ | N - RK | Not primary qualitative research | Not primary qualitative research | Not primary qualitative research |
| Impact of accountability, training, and human factors on the use of artificial intelligence in healthcare: exploring the perceptions of healthcare practitioners in the US | 2022 | Human factors in healthcare | Choudhury and Asan | Maybe – RK | Maybe – AF | N – AF | N - RK | Diagnostic AI not explicitly investigated | Diagnostic AI not explicitly investigated | Diagnostic AI not explicitly investigated |
| Artificial intelligence and the future of midwifery: what do midwives think about artificial intelligence? A qualitative study | 2022 | Health care for women international | Citil and Citil | Y - RK | Y - AF | N - JS | N - RK | Diagnostic AI not explicitly investigated | Diagnostic AI not explicitly investigated | Diagnostic AI not explicitly investigated |
| Radiographers’ knowledge, attitudes and expectations of artificial intelligence in medical imaging | 2022 | Radiography | Coakley et al. | Y - RK | Y - AF | N - SD | N - RK | Not primary qualitative research | Not primary qualitative research | Not primary qualitative research |
| Evaluation of European based radiographers’ knowledge and attitude towards implementation of artificial intelligence and radiography | 2022 | Insights into Imaging Supplement | Coakley et al. | Y - RK | Y - JS | N - JC | N - RK | Conference abstract | Conference abstract | Conference abstract |
| Artificial intelligence: radiologists’ expectations and opinions gleaned from a nationwide online survey | 2021 | La Radiologica Medica | Coppola et al. | Y - AF | N - RK | N - RK | N - DJ | Not primary qualitative research | Not primary qualitative research | Not primary qualitative research |
| The drivers of acceptance of artificial intelligence-powered care pathways among medical professionals: a web-based survey | 2022 | JMIR formative research | Cornelissen et al. | Y - JS | N - RK | N - RK | N - AF | Not primary qualitative research | Not primary qualitative research | Not primary qualitative research |
| Physician and staff attitudes towards implementation of artificial intelligence-assisted colonoscopy | 2022 | Gastroenterology Supplement | Coronel et al. | Y - RK | Y - AF | N - RK | N - AF | Conference abstract | Conference abstract | Conference abstract |
| Stakeholder perspectives on the use of artificial intelligence in medicine | 2022 | Value in Health | Crossnohere et al. | Y - RK | Y - RH | N - RK | N - AF | Conference abstract | Conference abstract | Conference abstract |
| Australian perspectives on artificial intelligence in medical imaging | 2022 | Journal of medical radiation sciences | Currie et al. | Maybe - JC | Y - RK | N- AF | N - RK | Not primary qualitative research | Not primary qualitative research | Not primary qualitative research |
| Artificial intelligence and health equity in primary care: a qualitative study with key stakeholders | 2023 | medRxiv | d’Elia et al. | N – RK | Maybe – AF | N – RK | N – AF | Not peer-reviewed | Not peer-reviewed | Not peer-reviewed |
| What is morally at stake when using algorithms to make medical diagnoses? Expanding the discussion beyond risks and harms | 2021 | Theoretical medicine and bioethics | De Boer et al. | Maybe – RK | N – RH | N- AF | N – RK | Not primary qualitative research | Not primary qualitative research | Not primary qualitative research |
| AI in breast screening mammography: breast screening readers’ perspectives | 2022 | Insights into imaging | De Vries et al | Y – RK | Y – SD | N- JS | N – RK | Not primary qualitative research | Not primary qualitative research | Not primary qualitative research |
| Implementing artificial intelligence in Canadian primary care: barriers and strategies identified through a national deliberative dialogue | 2023 | PLoS one | Darcel et al. | Maybe - RK | Maybe – AF | N – AF | N - RK | Diagnostic AI not explicitly investigated | Diagnostic AI not explicitly investigated | Diagnostic AI not explicitly investigated |
| Implementation of artificial intelligence in disease prediction and healthcare system- a survey | 2021 | IEEE | Deeba and Patil | Y - RK | Y - RH | N - RK | N - AF | Systematic review | Systematic review | Systematic review |
| AI-driven decision support systems and epistemic reliance: a qualitative study on obstetricians’ and midwives’ perspectives on integrating AI-driven CTG into clinical decision making | 2024 | BMC medical ethics | Dlugatch et al. | Maybe – AF | Maybe – RK | N – RK | N - AF | Diagnostic AI not explicitly investigated | Diagnostic AI not explicitly investigated | Diagnostic AI not explicitly investigated |
| Trustworthy artificial intelligence and ethical design: public perceptions of trustworthiness of an AI-based decision-support tool in the context of intrapartum care | 2023 | BMC medical ethics | Dlugatch et al. | Maybe – RK | Maybe – AF | N – RK | N - AF | Diagnostic AI not explicitly investigated | Diagnostic AI not explicitly investigated | Diagnostic AI not explicitly investigated |
| Feasibility and acceptability of novel colonscopy with computer-aided early diagnosis of bowel cancer | 2019 | United European gastroenterology journal | Dolwani et al. | Y - RH | N - RK | N - RK | N - DJ | Conference abstract | Conference abstract | Conference abstract |
| Medical students’ attitude towards artificial intelligence a multicentre survey | 2019 | European radiology | Dos Santos et al. | Y - AF | Y - RK | N- AF | N - RK | Not primary qualitative research | Not primary qualitative research | Not primary qualitative research |
| A national survey on the knowledge and perception of artificial intelligence in radiology | 2021 | Portugese journal of public health Supplement | Dumic-Cule et al. | Y - RK | Y - AF | N - JS | N - RK | Conference abstract | Conference abstract | Conference abstract |
| Knowledge and perception about artificial intelligence among medical students | 2023 | Journal of clinical and diagnostic research | Durai and Anu | Y - RK | Maybe - RH | N - RK | N - JS | Conference abstract | Conference abstract | Conference abstract |
| A survey on the use of artificial intelligence by clinicians in dentistry and oral and maxillofacial surgery | 2022 | Medicina | Eschert et al. | Y – RK | Y - JC | N - RK | N - AF | Not primary qualitative research | Not primary qualitative research | Not primary qualitative research |
| Use of AI-based tools for healthcare purposes: a survey study from consumers’ perspectives | 2020 | BMC medical informatics and decision making | Esmaeilzadeh | Y - RK | Y - AF | N- AF | N - RK | Not primary qualitative research | Not primary qualitative research | Not primary qualitative research |
| Patients’ perceptions toward human-artificial intelligence interaction in health care: experimental study | 2021 | Journal of medical internet research | Esmaeilzadeh et al. | Y - RK | Y - JC | N- AF | N - RK | Not primary qualitative research | Not primary qualitative research | Not primary qualitative research |
| Investigating the impacting factors for the healthcare professionals to adopt artificial intelligence-based medical diagnosis support systems (AIMDSS) | 2020 | Annals of operations research | Fan et al. | Y - RK | Y - RH | N- AF | N - RK | Not primary qualitative research | Not primary qualitative research | Not primary qualitative research |
| What is the future of artificial intelligence in obstetrics? A qualitative study among healthcare professionals | 2023 | BMJ open | Fischer et al. | Maybe – RK | Maybe – AF | N – RK | N - AF | Diagnostic AI not explicitly investigated | Diagnostic AI not explicitly investigated | Diagnostic AI not explicitly investigated |
| Drivers and social implications of artificial intelligence adoption in healthcare during the COVID-19 pandemic | 2021 | PloS One | Frank et al. | Maybe - RK | Y - AF | N - RK | N - AF | Not primary qualitative research | Not primary qualitative research | Not primary qualitative research |
| Acceptance, barriers, and facilitators to implementing artificial intelligence-based decision support systems in emergency departments: quantitative and qualitative evaluation | 2022 | JMIR formative research | Fujimori et al. | Maybe - RK | Y - AF | N - DJ | N - RK | Not primary qualitative research | Not primary qualitative research | Not primary qualitative research |
| Responsibility and decision-making authority in using clinical decision support systems: an empirical-ethical exploration of German prospective professionals’ preferences and concerns | 2023 | Journal of medical ethics | Funer et al. | Maybe – RK | Maybe – AF | N – RK | N - AF | Diagnostic AI not explicitly investigated | Diagnostic AI not explicitly investigated | Diagnostic AI not explicitly investigated |
| Exploring the experiences and views of doctors working with artificial intelligence in English healthcare; a qualitative study | 2023 | PloS One | Ganapathi et al. | Maybe - RK | Maybe – AF | N – AF | N - RK | Diagnostic AI not explicitly investigated | Diagnostic AI not explicitly investigated | Diagnostic AI not explicitly investigated |
| Lessons from the COVID-19 pandemic on the use of artificial intelligence in digital radiology: the submission of a survey to investigate the opinion of insiders | 2021 | Healthcare | Giansanti et al. | Y - RH | Y - RK | N - AF | N - RK | Not primary qualitative research | Not primary qualitative research | Not primary qualitative research |
| Professional implications of introducing artificial intelligence in healthcare: an evaluation using radiation medicine as a testing ground | 2019 | Journal of radiotherapy in practice | Gillan et al. | Y - RH | N - RK | N - RK | N - AF | Diagnostic AI not explicitly investigated | Diagnostic AI not explicitly investigated | Diagnostic AI not explicitly investigated |
| Artificial intelligence in digital pathology: what is the future? Part 2: an investigation on the insiders | 2021 | Healthcare | Giovagnoli et al. | Maybe - RH | Y - RK | N - RK | N - JC | Not primary qualitative research | Not primary qualitative research | Not primary qualitative research |
| Patient perceptions of the use of artificial intelligence in diagnostic radiology | 2021 | Journal of medical imaging and radiation oncology Supplement | Goh et al. | Y - RK | Y - RH | N - JS | N - RK | Conference abstract | Conference abstract | Conference abstract |
| Engaging stakeholders in a substantive and transparent way when implementing ethics in medical AI: a qualitative study | 2023 | Studies in health technology and informatics | Goirand et al. | Maybe – RK | Maybe – AF | N – AF | N - RK | Diagnostic AI not explicitly investigated | Diagnostic AI not explicitly investigated | Diagnostic AI not explicitly investigated |
| Implementation of an artificial intelligence algorithm for sepsis detection | 2020 | Revista brasileira de enfermagem | Goncalves et al. | Y - JC | Y - RK | N - RK | N - AF | Not primary qualitative research | Not primary qualitative research | Not primary qualitative research |
| Patients’ views on AI for risk prediction in shared decision-making for knee replacement surgery: qualitative interview study | 2023 | Journal of medical internet research | Gould et al. | N – RK | Maybe – AF | N – RK | N - AF | Diagnostic AI not explicitly investigated | Diagnostic AI not explicitly investigated | Diagnostic AI not explicitly investigated |
| Acceptance and use of a clinical decision support system in musculoskeletal pain disorders – the SupportPrim project | 2023 | BMC medical informatics and decision making | Granviken et al. | Maybe – RK | Maybe – AF | N – AF | N - RK | Diagnostic AI not explicitly investigated | Diagnostic AI not explicitly investigated | Diagnostic AI not explicitly investigated |
| Acceptance and perception of artificial intelligence usability in eye care (APPRAISE) for ophthalmologists: a multi-national perspective | 2022 | Frontiers in medicine | Gunasekeran et al. | Maybe - JC | Y - RK | N - RK | N - AF | Not primary qualitative research | Not primary qualitative research | Not primary qualitative research |
| Artificial intelligence and collaborative robots in healthcare: the perspective of healthcare professionals | 2023 | Transformation for sustainable business and management practices: exploring the spectrum of industry 5.0 | Gurce et al. | Maybe - AF | Maybe – RK | N – RK | N - AF | Diagnostic AI not explicitly investigated | Diagnostic AI not explicitly investigated | Diagnostic AI not explicitly investigated |
| Digital natives’ preferences on mobile artificial intelligence apps for skin cancer diagnostics: survey study | 2021 | JMIR mhealth and uhealth | Haggenmueller et al. | N - RK | Y - JC | N - RK | N - DJ | Not primary qualitative research | Not primary qualitative research | Not primary qualitative research |
| Defining user needs for a new sepsis risk decision support system in neonatal ICU settings through ethnography: user interviews and participatory design | 2019 | Human systems engineering and design | Harte et al. | N - RK | Y - RH | N - RK | N - AF | Diagnostic AI not explicitly investigated | Diagnostic AI not explicitly investigated | Diagnostic AI not explicitly investigated |
| Patients’ attitudes on the use of AI-based decision aid: a qualitative study | 2023 | Radiotherapy and oncology | Hasannejadasl et al. | Maybe – AF | Maybe – RK | N – AF | N - RK | Diagnostic AI not explicitly investigated | Diagnostic AI not explicitly investigated | Diagnostic AI not explicitly investigated |
| UK radiology trainees’ attitudes towards artificial intelligence in clinical radiology and its implementation in training and the curriculum | 2022 | Clinical radiology | Hashmi et al. | Y -RK | Y - AF | N - RK | N - JS | Conference abstract | Conference abstract | Conference abstract |
| Experiences regarding use and implementation of artificial intelligence-supported follow-up of atypical moles at a dermatological outpatient clinic: qualitative study | 2023 | JMIR Dermatology | Haugsten et al. | Y – RK | Y – AF | N – RK | N - AF | Diagnostic AI not explicitly investigated | Diagnostic AI not explicitly investigated | Diagnostic AI not explicitly investigated |
| Attitudes of anesthesiologists toward artificial intelligence in anesthesia: a multicenter, mixed qualitative-quantitative study | 2023 | Journal of clinical medicine | Henckert et al. | Maybe - AF | Maybe – RK | N – RK | N - AF | Diagnostic AI not explicitly investigated | Diagnostic AI not explicitly investigated | Diagnostic AI not explicitly investigated |
| Radiologist preferences for artificial intelligence-based decision support during screening mammography interpretation | 2022 | Journal of the American College of Radiology | Hendrix et al. | Y - RK | Y - AF | Y - DJ | N - RK |  | Not primary qualitative research | Not primary qualitative research |
| Artificial intelligence in breast cancer screening: primary care provider preferences | 2020 | Journal of the American Medical Informatics Association: JAMIA | Hendrix et al. | Y – JC | Y - RK | N - RK | N - AF | Not primary qualitative research | Not primary qualitative research | Not primary qualitative research |
| Study of patient and physician attitudes toward automated prognostic models for patients with metastatic cancer | 2023 | JCO clinical cancer informatics | Hildebrand et al. | Maybe – AF | N – RK | N – RK | N - AF | Diagnostic AI not explicitly investigated | Diagnostic AI not explicitly investigated | Diagnostic AI not explicitly investigated |
| Artificial intelligence for radiotherapy auto-contouring: current use, perceptions of and barriers to implementation | 2023 | Clinical oncology | Hindocha et al. | Maybe - RH | N - RK | N - RK | N - AF | Not primary qualitative research | Not primary qualitative research | Not primary qualitative research |
| Physician- and patient-elicited barriers and facilitators to implementation of a machine learnin-based screening tool for peripheral arterial disease: preimplementation study with physician and patient stakeholders | 2023 | JMIR cardio | Ho et al. | Maybe – RK | Maybe – AF | N – RK | N - AF | Diagnostic AI not explicitly investigated | Diagnostic AI not explicitly investigated | Diagnostic AI not explicitly investigated |
| Attitudes of optometrists towards artificial intelligence for the diagnosis of retinal disease: a cross-sectional mail-out survey | 2022 | Ophthalmic and physiological optics: the journal of the British College of Ophthalmic Opticians | Ho et al. | Maybe – JC | Y – RK | N – RK | N – AF | Not primary qualitative research | Not primary qualitative research | Not primary qualitative research |
| The impact of AI in the UK healthcare industry: a socio-technical system theory perspective | 2022 | CEUR Workshop Proceedings | Holdsworth and Zaghloul | Y- RK | Y – JS | N – RK | N – AF | Not peer-reviewed | Conference abstract | Not peer-reviewed |
| Attitudes and acceptance towards artificial intelligence in medical care | 2022 | Studies in health technology and informatics | Holzner et al. | Y – RK | Maybe – JC | N – RK | N – DJ | Diagnostic AI not explicitly investigated | Diagnostic AI not explicitly investigated | Diagnostic AI not explicitly investigated |
| Healthcare provider evaluation of machine learning-directed care: reactions to deployment on a randomised controlled study | 2023 | BMJ health and care informatics | Hong et al. | N – RH | Maybe – RK | N – RK | N – JS | Not primary qualitative research | Not primary qualitative research | Not primary qualitative research |
| Attitudes of the surgical team toward artificial intelligence in neurosurgery: an international two-stage cross-sectional survey | 2020 | World neurosurgery | Horsfall et al. | Maybe – JC | Y – RK | N – RK | N – SD | Not primary qualitative research | Not primary qualitative research | Not primary qualitative research |
| An international survey on AI in radiology in 1041 radiologists and radiology residents part 1: fear of replacement, knowledge and attitude | 2021 | European radiology | Huisman et al. | Y – RK | Y – AF | N – RK | N – AF | Not primary qualitative research | Not primary qualitative research | Not primary qualitative research |
| Perception of the impact of artificial intelligence in the decision-making processes of public healthcare professionals | 2022 | Journal of environmental and public health | Ibrahim et al. | Y – RK | Y – AF | N – RK | N – AF | Diagnostic AI not explicitly investigated | Diagnostic AI not explicitly investigated | Diagnostic AI not explicitly investigated |
| The adoption of artificial intelligence in health care and social services in Australia: findings from a methodologically innovative national survey of values and attitudes (the AVA-AI study) | 2022 | Journal of medical internet research | Isbanner et al. | Maybe – JC | Y – RK | N – RK | N – AF | Not primary qualitative research | Not primary qualitative research | Not primary qualitative research |
| Over-detection of melanoma-suspect lesions by a CE-certified smartphone app: performance in comparison to dermatologists, 2D and 3D convolutional neural networks in a prospective data set of 1204 pigmented skin lesiosn involving patients’ perception | 2022 | Cancers | Jahn et al. | Y – RK | N – RH | N – RK | N – AF | Not primary qualitative research | Not primary qualitative research | Not primary qualitative research |
| Technology acceptance of a machine-learning algorithm predicting delirium in a clinical setting: a mixed-methods study | 2021 | Journal of medical systems | Jauk et al. | N – RK | Y – RH | N – RK | N – AF | Diagnostic AI not explicitly investigated | Diagnostic AI not explicitly investigated | Diagnostic AI not explicitly investigated |
| Evaluation framework for successful artificial intelligence-enabled clinical decision support systems; mixed methods study | 2021 | Journal of medical internet research | Ji et al. | Y – RH | N – RK | N – RK | N – AF | Not primary qualitative research | Not primary qualitative research | Not primary qualitative research |
| The impact of cultural embeddedness on the implementation of an artificial intelligence program at triage: a qualitative study | 2022 | Journal of transcultural nursing | Jordan et al. | Y – RK | Y – RH | N – RK | N – JC | Diagnostic AI not explicitly investigated | Diagnostic AI not explicitly investigated | Diagnostic AI not explicitly investigated |
| Machine learning-based and rule-based sepsis risk prediction tools: a qualitative study of implementation challenges and approaches | 2020 | Journal of General Internal Medicine | Joshi et al. | Y- RK | Y – RH | N – RK | N – AF | Conference abstract | Not peer-reviewed | Conference abstract |
| Modeling conceptual framework for implementing barriers of AI in public healthcare for improving operational excellence: experiences from developing countries | 2022 | Sustainability | Joshi et al. | Maybe – RK | Y – RH | N – AF | N – RK | Systematic review | Systematic review | Systematic review |
| Trust in artificial intelligence for medical diagnoses | 2020 | Prog Brain Res | Juravle et al. | Maybe – RK | Y – AF | N – RK | N – JS | Not primary qualitative research | Not primary qualitative research | Not primary qualitative research |
| Augmenting medical diagnosis decisions? An investigation into physicians’ decision-making process with artificial intelligence | 2021 | Information systemas research | Jussupow et al. | Y – RH | Y – RK | N – RH | N – RK | Diagnostic AI not explicitly investigated | Diagnostic AI not explicitly investigated | Diagnostic AI not explicitly investigated |
| Artificial intelligence in skin cancer diagnostics: the patients’ perspective | 2020 | Frontiers in medicine | Jutzi et al. | Y – RK | Y – SD | N – RK | N – RH | Not primary qualitative research | Not primary qualitative research | Not primary qualitative research |
| Opinions of UK gastroenterology consultants in the application of artificial intelligence in endoscopy | 2021 | Gut | Kader et al. | Y – RK | Y – AF | N – DJ | N- RK | Conference abstract | Conference abstract | Conference abstract |
| Survey on the perceptions of UK gastroenterologists and endoscopists to artificial intelligence | 2022 | Frontline gastroenterology | Kader et al. | Y – JS | Y – RK | N – RH | N – RK | Not primary qualitative research | Not primary qualitative research | Not primary qualitative research |
| Exploring physician perspectives on using real-world care data for the development of artificial intelligence-based technologies in health care: qualitative study | 2022 | JMIR formative research | Kamradt et al. | Y – RK | Y – SD | N – JS | N – RK | Diagnostic AI not explicitly investigated | Diagnostic AI not explicitly investigated | Diagnostic AI not explicitly investigated |
| A scenario-based approach for designing a stroke clinical decision support system | 2012 | IADIS | Kao et al. | Y – RK | Y – RH | N – AF | N – RK | Not primary qualitative research | Not primary qualitative research | Not primary qualitative research |
| Integrating human factors into trustworthy AI for healthcare | 2023 | ACM International Conference Proceeding Series | Karimian et al. | Maybe – AF | Maybe – RK | N – AF | N – RK | Diagnostic AI not explicitly investigated | Diagnostic AI not explicitly investigated | Diagnostic AI not explicitly investigated |
| The impact of artificial intelligence in radiology: as perceived by medical students | 2020 | Russian electronic journal of radiology | Kasetti and Botchu | Y – AF | Y – RK | N – RK | N – AF | Not primary qualitative research | Not primary qualitative research | Not primary qualitative research |
| Academic machine learning researchers’ ethical perspectives on algorithm development for health care: a qualitative study | 2023 | Journal of the American medical informatics association: JAMIA | Kasun et al. | Maybe – RK | Maybe – AF | N – AF | N – RK | Diagnostic AI not explicitly investigated | Diagnostic AI not explicitly investigated | Diagnostic AI not explicitly investigated |
| A survey of extant organizational and computational setups for deploying predictive models in health systems | 2021 | Journal of the American medical informatics association: JAMIA | Kashyap et al. | Maybe – RH | N – RK | N – RK | N – AF | Diagnostic AI not explicitly investigated | Diagnostic AI not explicitly investigated | Diagnostic AI not explicitly investigated |
| Feasibility and patient acceptability of a novel artificial intelligence-based screening model for diabetic retinopathy at endocrinology outpatient servicesL a pilot study | 2018 | Scientific reports | Keel et al. | Y – JS | N – RK | N – RK | N – AF | Not primary qualitative research | Not primary qualitative research | Not primary qualitative research |
| Canadian oncology residents’ knowledge of and attitudes towards artificial intelligence and machine learning | 2022 | Radiotherapy and oncology Supplement | Kennedy et al. | Y – AF | Y – RK | N – RK | N – SD | Conference abstract | Conference abstract | Conference abstract |
| Choosing human over AI doctors? How comparative trust associations and knowledge relate to risk and benefit perceptions of AI in healthcare | 2023 | Risk analysis: an official publication of the Society for Risk Analysis | Kerstan et al. | N – RK | Maybe – AF | N – AF | N – RK | Not primary qualitative research | Not primary qualitative research | Not primary qualitative research |
| How does the radiology community discuss the benefits and limitations of artificial intelligence for their work? A systematic discourse analysis | 2021 | European journal of radiology | Kim et al. | Maybe – RK | Maybe – RH | N – AF | N – RK | Not primary qualitative research | Not primary qualitative research | Not primary qualitative research |
| The facilitators and barriers of the implementation of a clinical decision support system for breast cancer multidisciplinary team meetings – an interview study | 2024 | Cancers | Koco et al. | Maybe – AF | Maybe – RK | N – RK | N – AF | Diagnostic AI not explicitly investigated | Diagnostic AI not explicitly investigated | Diagnostic AI not explicitly investigated |
| User testing of a diagnostic decision support system with machine-assisted chart review to facilitate clinical genomic diagnosis | 2021 | BMJ health and care informatics | Kulchak Rahm et al. | Y – RK | Y – JS | N – RK | N – AF | Not primary qualitative research | Not primary qualitative research | Not primary qualitative research |
| Responsible artificial intelligence (AI) for value formation and market performance in healthcare: the mediating role of patient’s cognitive engagement | 2021 | Information systems frontiers | Kumar et al. | Y – RK | Y – AF | N – JS | N – RK | Diagnostic AI not explicitly investigated | Diagnostic AI not explicitly investigated | Diagnostic AI not explicitly investigated |
| The impact of cultural dimensions of clinicians on the adoption of artificial intelligence in healthcare | 2022 | Journal of association of physicians of India | Krishnamoorthy et al. | Y – RK | Y – AF | N – AF | N – RK | Not primary qualitative research | Not primary qualitative research | Not primary qualitative research |
| Influence of AI ethics awareness, attitude, anxiety, and self-efficacy on nursing students’ behavioural intentions | 2022 | BMC nursing | Kwak at al. | N – RK | Y – AF | N – AF | N – RK | Not primary qualitative research | Not primary qualitative research | Not primary qualitative research |
| Perceptions of artificial intelligence in healthcare: findings from a qualitative survey study among actors in France | 2020 | Journal of translational medicine | Lai et al. | Y – RK | Y – AF | N – AF | N – RK | Diagnostic AI not explicitly investigated | Diagnostic AI not explicitly investigated | Diagnostic AI not explicitly investigated |
| Stuck in translation: stakeholder perspectives on impedeiments to responsible digital health | 2023 | Frontiers in digital health | Landers et al. | N – RK | Maybe – AF | N – AF | N – RK | Diagnostic AI not explicitly investigated | Diagnostic AI not explicitly investigated | Diagnostic AI not explicitly investigated |
| Machine learning driven tools in orthopaedics and spine surgery: hype or reality? Applications and perception of 31 physician opinions | 2021 | Seminars in spine surgery | Lans et al. | Y – RK | N – AF | N – AF | N – RK | Not primary qualitative research | Systematic review | Systematic review |
| Nurse leaders’ and digital service developers’ perceptions of the future role of artificial intelligence in specialised medical care: an interview study | 2022 | Journal of nursing management | Laukka et al. | Y – JS | N – RK | N – AF | N – RK | Diagnostic AI not explicitly investigated | Diagnostic AI not explicitly investigated | Diagnostic AI not explicitly investigated |
| Factors facilitating the acceptance of diagnostic robots in healthcare: a survey | 2022 | IEEE | Laursen et al. | N – RH | Y – RK | N – JC | N – RK | Not primary qualitative research | Not primary qualitative research | Not primary qualitative research |
| Perception and expectations towards artificial intelligence in capsule endoscopy | 2021 | United European Gastroenterology Journal | Leenhardt et al. | Y – JS | Y – RK | N – RK | N – SD | Conference abstract | Conference abstract | Conference abstract |
| Use and control of artificial intelligence in patients across the medical workflow: single-centre questionnaire study of patient perspectives | 2021 | Journal of medical internet research | Lennartz et al. | Maybe – JS | Y – RK | N – AF | N – RK | Not primary qualitative research | Not primary qualitative research | Not primary qualitative research |
| Women’s attitudes to the use of AI image readers: a case study from a national breast screening programme | 2021 | BMH Health Care Informatics | Lennox-Chhugani et al. | Y – RK | Y – JC | N – RH | N – RK | Not primary qualitative research | Not primary qualitative research | Not primary qualitative research |
| Clinical decision support software for management of chronic heart failure: development and evaluation | 2006 | Computers in biology and medicine | Leslie et al. | Y – RH | N – RK | N – RK | N – AF | Diagnostic AI not explicitly investigated | Diagnostic AI not explicitly investigated | Diagnostic AI not explicitly investigated |
| Perceptions of Canadian vascular surgeons toward artificial intelligence and machine learning | 2022 | Journal of vascular surgery cases and innovative techniques | Li et al. | Maybe – JC | N – RK | N – RK | N – AF | Not primary qualitative research | Not primary qualitative research | Not primary qualitative research |
| Understanding medical students’ perceptions of and behavioural intentions toward learning artificial intelligence: a survey study | 2022 | International journal of environmental research and public health | Li et al. | Maybe – JC | Y – RK | N – RK | N – AF | Not primary qualitative research | Not primary qualitative research | Not primary qualitative research |
| Assessing usability and clinical utility of GLANCE, an artificial intelligence-based tool for predicting glaucoma visual field progression | 2022 | Investigative ophthalmology and visual science | Lieu et al. | N – RH | Maybe – RK | N – RK | N – AF | Conference abstract | Conference abstract | Conference abstract |
| Perceptions of the use of artificial intelligence in the diagnosis of skin cancer: an outpatient survey | 2021 | Clinical and experimental dermatology | Lim et al. | Y – SD | Y – SD | N – RK | N – AF | Not primary qualitative research | Not primary qualitative research | Not primary qualitative research |
| Revolutionising dental technologies: a qualitative study on dental technicians’ perceptions of artificial intelligence integration | 2023 | BMC oral health | Lin et al. | Maybe – AF | N – RK | N – AF | N – RK | Diagnostic AI not explicitly investigated | Diagnostic AI not explicitly investigated | Diagnostic AI not explicitly investigated |
| Adoption of a clinical decision support system to promote judicious use of antibiotics for acute respiratory infections in primary care | 2012 | International journal of medical informatics | Litvin et al. | N – RH | Y – RK | N – RK | N – DJ | Diagnostic AI not explicitly investigated | Diagnostic AI not explicitly investigated | Diagnostic AI not explicitly investigated |
| Does AI Explainability affect physicians’ intention to use AI? | 2022 | International journal of medical informatics | Liu et al. | Y – RK | Y – DJ | N – RK | N – AF | Not primary qualitative research | Not primary qualitative research | Not primary qualitative research |
| Discrepancy between perceptions and acceptance of clinical decision support systems: implementation of artificial intelligence for vancomycin dosing | 2023 | BMC medical informatics and decision making | Liu et al. | Maybe – RK | N – AF | N – AF | N – RK | Diagnostic AI not explicitly investigated | Diagnostic AI not explicitly investigated | Diagnostic AI not explicitly investigated |
| Artificial intelligence in primary health care: perceptions, issues and challenges | 2019 | Yearbook of medical informatics | Liyanage et al. | Maybe – JC | Y – RK | N – RK | N – AF | Diagnostic AI not explicitly investigated | Diagnostic AI not explicitly investigated | Diagnostic AI not explicitly investigated |
| Resistance to medical artificial intelligence | 2019 | Journal of consumer research | Longoni et al. | Y – RK | Maybe – RH | N – RK | N – AF | Not primary qualitative research | Diagnostic AI not explicitly investigated | Not primary qualitative research |
| Future medical artificial intelligence application requirements and expectations of physicians in German university hospitals: web-based survey | 2021 | Journal of medical internet research | Maassen et al. | Y – AF | Y – RK | N – RK | N – AF | Not primary qualitative research | Diagnostic AI not explicitly investigated | Not primary qualitative research |
| Clientele perception and trust on artificial intelligence integrated health services | 2022 | Industry 4.0 and intelligent business analytics for healthcare | Magpantay | Y – RK | N – AF | N – RK | N – AF | Commentary/editorial | Commentary/editorial | Commentary/editorial |
| Understanding patient views and acceptability of predictive software in osteoporosis identification | 2023 | Radiography | Manning et al. | Maybe – RK | Maybe – AF | N – AF | N - RK | Diagnostic AI not explicitly investigated | Diagnostic AI not explicitly investigated | Diagnostic AI not explicitly investigated |
| People’s perceptions of AI utilization in the context of COVID-19 | 2021 | Diversity, Divergence, Dialogue | Markazi and Walters | Y – RK | Y – RH | N – RK | N – JS | Conference abstract | Conference abstract | Conference abstract |
| A healthy debate: exploring the views of medical doctors on the ethics of artificial intelligence | 2021 | Artificial intelligence in medicine | Martinho et al. | Y – AF | N – RK | N – AF | N – RK | Diagnostic AI not explicitly investigated | Diagnostic AI not explicitly investigated | Diagnostic AI not explicitly investigated |
| Out with AI, in with the psychiatrist: a preference for human-derived clinical decision support in depression care | 2023 | Translational psychiatry | Maslej, M et al. | Maybe – RK | Maybe – AF | N – RK | N – AF | Diagnostic AI not explicitly investigated | Diagnostic AI not explicitly investigated | Diagnostic AI not explicitly investigated |
| Clinician preimplementation perspectives of a decision-support tool for the prediction of cardiac arrhythmia based on machine learning: near-live feasibility and qualitative study | 2021 | JMIR human factors | Matthiesen et al. | N – RK | Y – RH | N – RH | N – RK | Diagnostic AI not explicitly investigated | Diagnostic AI not explicitly investigated | Diagnostic AI not explicitly investigated |
| What’s in an explanation?: a grounded theory study to understand clinicians views on Explainability in healthcare machine learning | 2021 | OSF registry | McCradden | Y – RK | Maybe – RH | N- RK | N – AF | Study protocol | Study protocol | Study protocol |
| Ethical concerns around use of artificial intelligence in health care research from the perspective of patients with meningioma, caregivers and health care providers: a qualitative study | 2020 | CMAJ open | McCradden | Y – RK | Y – DJ | N – AF | N – RK | Diagnostic AI not explicitly investigated | Diagnostic AI not explicitly investigated | Diagnostic AI not explicitly investigated |
| The use of artificial intelligence in medical imaging: a nationwide pilot survey of trainees in Saudi Arabia | 2022 | Clinics and practice | Mirza et al. | Maybe – JC | Y – RK | N – RK | N – AF | Not primary qualitative research | Not primary qualitative research | Not primary qualitative research |
| Assessing barriers to implementation of machine learning and artificial intelligence based tools in critical care: web-based survey study | 2023 | JMIR perioperative medicine | Mlodzinski et al. | N – AF | Maybe – RK | N – RK | N – RH | Not primary qualitative research | Not primary qualitative research | Not primary qualitative research |
| Enhancing artificial intelligence-doctor collaboration for computer-aided diagnosis in colonoscopy through improved digital literacy | 2023 | Dig Liver Dis | Mori et al. | Maybe – RK | Maybe – AF | N – RK | N – AF | Not primary qualitative research | Commentary/editorial | Not primary qualitative research |
| Artificial intelligence and the NHS: a qualitative exploration of the factors influencing adoption | 2020 | BMJ Leader Supplement | Morrison | Y – RK | Y – AF | N – RK | N – SD | Conference abstract | Conference abstract | Conference abstract |
| Increased automation and artificial intelligence in radiation therapy clinical practice: a qualitative exploration of radiation therapist predictions | 2019 | Journal of medical imaging and radiation sciences | Moyo et al. | Y – RH | N – RK | N – AF | N – RK | Conference abstract | Conference abstract | Conference abstract |
| Perceptions of artificial intelligence use in primary care: a qualitative study with providers and staff of Ontario community health centres | 2023 | Journal of the American Board of Family Medicine | Nash et al. | Y – RK | Maybe – AF | N – RK | N – AF | Diagnostic AI not explicitly investigated | Diagnostic AI not explicitly investigated | Diagnostic AI not explicitly investigated |
| Innovation in healthcare: leadership perceptions about the innovation characteristics of artificial intelligence- a qualitative interview study with healthcare leaders in Sweden | 2023 | Implementation science communications | Neher et al. | Maybe - AF | Maybe – RK | N – RK | N - AF | Diagnostic AI not explicitly investigated | Diagnostic AI not explicitly investigated | Diagnostic AI not explicitly investigated |
| A survey of knowledge, perceptions, and barriers on the use of artificial intelligence in patients with inflammatory bowel disease in the US and UK | 2020 | American Journal of Gastroenterology | Nigam et al. | Y - RK | Y - AF | N - DJ | N - RK | Conference abstract | Conference abstract | Conference abstract |
| How the UK public views the use of diagnostic decision aids by physicians: a vignette-based experiment | 2023 | Journal of the American medical informatics association: JAMIA | Nurek and Kostopoulou | Y - RK | Y - RH | N - JS | N - RK | Not primary qualitative research | Not primary qualitative research | Not primary qualitative research |
| Physician confidence in artificial intelligence: an online mobile survey | 2019 | Journal of medical internet research | Oh et al. | Y - RK | Y - JS | N - RK | N - JS | Not primary qualitative research | Not primary qualitative research | Not primary qualitative research |
| Patients’ views on the implementation of artificial intelligence in radiology: development and validation of a standardized questionnaire | 2019 | European radiology | Ongena et al. | Y - RK | Y - JS | N - RK | N - RH | Not primary qualitative research | Not primary qualitative research | Not primary qualitative research |
| Attitudes of patients and their relatives towards artificial intelligence in neurosurgery | 2020 | World neurosurgery | Palmisciano et al. | Y - RK | N - SD | N - RK | N - DJ | Not primary qualitative research | Not primary qualitative research | Not primary qualitative research |
| The value of artificial intelligence in laboratory medicine | 2020 | American journal of clinical pathology | Paranjape et al. | Y - RK | Y - JC | N - JS | N - RK | Not primary qualitative research | Not primary qualitative research | Not primary qualitative research |
| The use and future perspective of artificial intelligence- a survey among German surgeons | 2022 | Frontiers in public health | Pecqueux et al. | Y - RK | Y - AF | N - AF | N - RK | Not primary qualitative research | Not primary qualitative research | Not primary qualitative research |
| Knowledge, attitudes, and practices towards artificial intelligence among young pediatricians: a nationwide survey in France | 2022 | Frontiers in pediatrics | Perrier et al. | Y - RK | Y - AF | N - AF | N - RK | Not primary qualitative research | Not primary qualitative research | Not primary qualitative research |
| Women’s perceptions and attitudes to the use of AI in breast cancer screening: a survey in a cancer referral centre | 2022 | The British journal of radiology | Pesapane et al. | Y - RK | Y - JS | N - AF | N - RK | Not primary qualitative research | Not primary qualitative research | Not primary qualitative research |
| Challenges to implementing artificial intelligence in healthcare: a qualitative interview study with healthcare leaders in Sweden | 2022 | BMC health services research | Petersson et al. | Y - AF | N - RK | N - JS | N - RK | Diagnostic AI not explicitly investigated | Diagnostic AI not explicitly investigated | Diagnostic AI not explicitly investigated |
| Factors of adoption of artificial intelligence (AI) and internet of medical things (IoT) amongst healthcare workers: a descriptive analysis | 2022 | International journal of systematic innovations | Sikarwar et al. | Y - RH | N - RK | N - RK | N - JC | Diagnostic AI not explicitly investigated | Diagnostic AI not explicitly investigated | Diagnostic AI not explicitly investigated |
| Acceptability of artificial intelligence among Indian dermatologists | 2022 | Indian journal of dermatology, venereology and leprology | Pangti et al. | N - RH | Y - RK | N - RK | N - DJ | Not primary qualitative research | Not primary qualitative research | Not primary qualitative research |
| What should medical students know about artificial intelligence in medicine? | 2019 | Journal of educational evaluation for health professions | Park et al. | Maybe - RK | Maybe - AF | N - AF | N - RK | Not primary qualitative research | Not primary qualitative research | Not primary qualitative research |
| Attitudes toward artificial intelligence within dermatopathology: an international online survey | 2020 | Frontiers in medicine | Polesie et al. | Y - DJ | Y - RK | N - AF | N - RK | Not primary qualitative research | Not primary qualitative research | Not primary qualitative research |
| Evaluating the clinical feasibility of an artificial intelligence-powered, web-based clinical decision support system for the treatment of depression in adults: longitudinal feasibility study | 2021 | JMIR formative research | Popescu et al. | N - RK | Y - RH | N - AF | N - RK | Diagnostic AI not explicitly investigated | Diagnostic AI not explicitly investigated | Diagnostic AI not explicitly investigated |
| Eliciting user decision requirements for designing computerised diagnostic support for family physicians | 2016 | Journal of cognitive engineering and decision making | Porat et al. | N - RK | Y - RH | N - AF | N - RK | Diagnostic AI not explicitly investigated | Diagnostic AI not explicitly investigated | Diagnostic AI not explicitly investigated |
| Exploration of exposure to artificial intelligence in undergraduate medical education: a Canadian cross-sectional mixed-methods study | 2022 | BMC medical education | Pucchio et al. | N - RK | Maybe - AF | N - AF | N - JS | Diagnostic AI not explicitly investigated | Diagnostic AI not explicitly investigated | Diagnostic AI not explicitly investigated |
| Saudi radiology personnels’ perceptions of artificial intelligence implementation: a cross-sectional study | 2021 | Journal of multidisciplinary healthcare | Qurashi et al. | Y - RK | Y - AF | N - RK | N - JS | Not primary qualitative research | Not primary qualitative research | Not primary qualitative research |
| An insight into the current perceptions of UK radiographers on the future impact of AI on the profession: a cross-sectional survey | 2022 | Journal of medical imaging and radiation science | Rainey et al. | Y - RK | Y - JS | N - RK | N - RH | Not primary qualitative research | Not primary qualitative research | Not primary qualitative research |
| UK reporting radiographers’ perceptions of AI in radiographic image interpretation – current perspectives and future developments | 2022 | Radiography | Rainey et al. | Y - RK | Y - SD | N - RK | N - RH | Not primary qualitative research | Not primary qualitative research | Not primary qualitative research |
| The use of digital pathology and artificial intelligence in histopathological diagnostic assessment of prostate cancer: a survey of prostate cancer UK supporters | 2022 | Diagnostics | Rakovic et al. | Y - RK | Y - AF | N - SD | N - RK | Not primary qualitative research | Not primary qualitative research | Not primary qualitative research |
| Parental perceptions on use of artificial intelligence in pediatric acute care | 2023 | Academic pediatrics | Ramgopal et al. | Maybe - JC | Y - RK | N - RK | N - SD | Not primary qualitative research | Not primary qualitative research | Not primary qualitative research |
| Physicians’ attitudes towards artificial intelligence in medicine, their expectations and concernsL an online mobile survey | 2021 | Malaysian journal of public health medicine | Reffien et al. | Y - RK | Maybe - JC | N - RK | N - RH | Not primary qualitative research | Not primary qualitative research | Not primary qualitative research |
| Artificial intelligence-integrated approaches in ophthalmology: a qualitative pilot study of provider understanding and adoption of AI | 2022 | Investigative ophthalmology and visual science | Robinson et al. | Maybe - JS | Y - RK | N - RK | N - JC | Conference abstract | Conference abstract | Conference abstract |
| Non-user physician perspectives about an oncology clinical decision-support system: a qualitative study | 2020 | Journal of clinical oncology | Rocha et al. | Y - RH | N - RK | N - RK | N - DJ | Conference abstract | Conference abstract | Conference abstract |
| Public perceptions and implementation considerations on the use of artificial intelligence in health | 2022 | Journal of evaluation in clinical practice | Romero and Young | Y - RH | Y- RK | N - AF | N - RK | Diagnostic AI not explicitly investigated | Diagnostic AI not explicitly investigated | Diagnostic AI not explicitly investigated |
| A lesson in implementation: a pre-post study of providers’ experience with artificial intelligence-based clinical decision support | 2020 | International journal of medical informatics | Romero-Brufau et al. | Y - RH | N - RK | N - DJ | N - RK | Not primary qualitative research | Not primary qualitative research | Not primary qualitative research |
| A computerised clinical decision support system for diagnosing children’s brain tumours using functional imaging and machine learning | 2022 | Neuro-oncology | Rose et al. | Y - RH | Y - RK | N - RK | N - JS | Conference abstract | Conference abstract | Conference abstract |
| Competencies for the use of artificial intelligence-based tools by health care professionals | 2022 | Academic medicine: journal of the association of American medical colleges | Russell et al. | Y - AF | N - RK | N - AF | N - RK | Diagnostic AI not explicitly investigated | Diagnostic AI not explicitly investigated | Diagnostic AI not explicitly investigated |
| Artificial intelligence: the opinions of radiographers and radiation therapists in Ireland | 2021 | Radiography | Ryan et al. | Maybe - JC | Y - RK | N - RK | N - AF | Not primary qualitative research | Not primary qualitative research | Not primary qualitative research |
| “Nothing works without the doctor”: physicians’ perception of clinical decision-making and artificial intelligence | 2022 | Frontiers in medicine | Samhammer et al. | Y - RH | N - RK | N - AF | N - RK | Diagnostic AI not explicitly investigated | Diagnostic AI not explicitly investigated | Diagnostic AI not explicitly investigated |
| Awareness and knowledge about artificial intelligence in healthcare among doctors – a survey | 2020 | European journal of molecular nad clinical medicine | Samyuktha et al. | Y - RK | Maybe - JC | N - RK | N - AF | Not primary qualitative research | Not primary qualitative research | Not primary qualitative research |
| Physician perspectives on integration of artificial intelligence into diagnostic pathology | 2019 | NPJ digital medicine | Sarwar et al. | Y - AF | Y - RK | N - RK | N - AF | Not primary qualitative research | Not primary qualitative research | Not primary qualitative research |
| Barriers and opportunities regarding implementation of a machine learning-based acute heart failure risk stratification tool in the emergency department | 2022 | Diagnostics | Sax et al. | N - RK | Y - RH | N - AF | N - RK | Diagnostic AI not explicitly investigated | Diagnostic AI not explicitly investigated | Diagnostic AI not explicitly investigated |
| Artificial intelligence assisted opportunistic screening for referable diabetic retinopathy: from algorithm to real world application | 2019 | Clinical and experimental ophthalmology | Scheetz et al. | Y - RH | Y - RK | N - RK | N - DJ | Conference abstract | Conference abstract | Conference abstract |
| A survey of clinicians on the use of artificial intelligence in ophthalmology, dermatology, radiology and radiation oncology | 2021 | Scientific reports | Scheetz et al. | Maybe - JC | Y - RK | N - RK | N - AF | Not primary qualitative research | Not primary qualitative research | Not primary qualitative research |
| Implementing artificial intelligence in clinical practice: a mixed-method study of barriers and facilitators | 2022 | Journal of medical artificial intelligence | Schouten et al. | Y - RK | Y - AF | N - DJ | N - RK | Diagnostic AI not explicitly investigated | Diagnostic AI not explicitly investigated | Diagnostic AI not explicitly investigated |
| Clinical decision support with arden syntax in routine clinical practice | 2015 | Artif intel med | Schuh et al. | Maybe - RH | Maybe - RH | N - RK | N - JS | Conference abstract | Conference abstract | Conference abstract |
| Factors influencing clinician trust in predictive clinical decision support systems for in-hospital deterioration: qualitative descriptive study | 2022 | JMIR human factors | Schwartz et al. | Y - RH | N - RK | N - JS | N - RK | Diagnostic AI not explicitly investigated | Diagnostic AI not explicitly investigated | Diagnostic AI not explicitly investigated |
| Clinicians’ perceptions of artificial intelligence: focus on workload, risk, trust, clinical decision making, and clinical integration | 2023 | Healthcare | Shamszare and Choudhury | Maybe – RK | N – AF | N – RK | N - AF | Not primary qualitative research | Not primary qualitative research | Not primary qualitative research |
| Artificial intelligence in paediatric radiology: international survey of health care professionals’ opinions | 2022 | Pediatric radiology | Shelmerdine et al. | Y – RK | Y - AF | N - AF | N - RK | Not primary qualitative research | Not primary qualitative research | Not primary qualitative research |
| Web-based study on Chinese dermatologists’ attitudes towards artificial intelligence | 2020 | Annals of translational medicine | Shen et al. | Y - RK | Y - JC | N - AF | N - RK | Not primary qualitative research | Not primary qualitative research | Not primary qualitative research |
| Exploring the influence of decision style on decision support system acceptance by GPs | 2009 | AISEL | Shibl et al. | Y - RK | Y - AF | N - RK | N - SD | Conference abstract | Conference abstract | Conference abstract |
| Predicting GPs’ engagement with artificial intelligence | 2018 | British journal of health care management | Sola et al. | N - RH | Y - RK | N - JS | N - RK | Not primary qualitative research | Not primary qualitative research | Not primary qualitative research |
| The application of artificial intelligence: perceptions from healthcare professionals | 2023 | Health and technology | Sonawane et al. | Maybe – AF | Maybe - RK | N – AF | N - RK | Diagnostic AI not explicitly investigated | Diagnostic AI not explicitly investigated | Diagnostic AI not explicitly investigated |
| Public perceptions of artificial intelligence and robotics in medicine | 2020 | Journal of endourology | Stai et al. | Y - RK | Y - AF | N - AF | N - RK | Not primary qualitative research | Not primary qualitative research | Not primary qualitative research |
| Attitudes towards artificial intelligence in emergency medicine | 2023 | Emergency medicine Australasia | Stewart et al. | Maybe – RK | Maybe - AF | N – RK | N - AF | Diagnostic AI not explicitly investigated | Diagnostic AI not explicitly investigated | Diagnostic AI not explicitly investigated |
| Artificial intelligence: the newest member of the liver transplant evaluation team? | 2021 | Hepatology Supplement | Strauss et al. | Y - RK | Y - AF | N - AF | N - RK | Conference abstract | Conference abstract | Conference abstract |
| Parental attitudes toward artificial intelligence-driven precision medicine technologies in pediatric healthcare | 2020 | Children | Sisk et al. | Y - RK | Y - AF | N - DJ | N - RK | Not primary qualitative research | Not primary qualitative research | Not primary qualitative research |
| Stakeholder perceptions of the safety and assurance of artificial intelligence in healthcare | 2022 | Safety science | Sujan et al. | Y - AF | Y - RK | N - RK | N - AF | Diagnostic AI not explicitly investigated | Diagnostic AI not explicitly investigated | Diagnostic AI not explicitly investigated |
| Knowledge, attitudes, and perceptions regarding the future of artificial intelligence in oral radiology in India: a survey | 2020 | Imaging science in dentistry | Sur et al. | Y - RK | Y - DJ | N - JC | N - RK | Not primary qualitative research | Not primary qualitative research | Not primary qualitative research |
| Knowledge, attitude and practice of artificial intelligence among doctors and medical students in SyriaL a cross-sectional online survey | 2022 | Frontiers in artificial intelligence | Swed et al. | Y - JC | Y - RK | N - AF | N - RK | Not primary qualitative research | Not primary qualitative research | Not primary qualitative research |
| Artificial intelligence and clinical decision support systems or automated interpreters: what characteristics are expected by French general practitioners? | 2022 | Studies in health technology and informatics | Tabla et al. | Y – RK | Y - RH | N - JS | N - RK | Not primary qualitative research | Not primary qualitative research | Not primary qualitative research |
| Evaluation of radiologist’s knowledge about the artificial intelligence in diagnostic radiology: a survey-based study | 2020 | Acta radiologica open | Tajaldeen and Alghamdi | Y - RK | Y - AF | N - RK | N - AF | Not primary qualitative research | Not primary qualitative research | Not primary qualitative research |
| Acceptance of the use of artificial intelligence in medicine among Japan’s doctors and the public: a questionnaire study | 2022 | JMIR human factors | Tamori et al. | Y - JS | Y - RK | N - RK | N - JS | Not primary qualitative research | Not primary qualitative research | Not primary qualitative research |
| Roles and competencies of doctors in artificial intelligence implementation: qualitative analysis through physician interviews | 2023 | JMIR formative research | Tanaka et al. | Maybe – RK | Maybe – AF | N - AF | N - RK | Diagnostic AI not explicitly investigated | Diagnostic AI not explicitly investigated | Diagnostic AI not explicitly investigated |
| Is primary health care ready for artificial intelligence? Stakeholder perspectives: worth the risk as long as you do it well | 2022 | Annals of family medicine | Terry et al. | Maybe – RK | Maybe – AF | N – RK | N - AF | Conference abstract | Diagnostic AI not explicitly investigated | Conference abstract |
| Is primary health care ready for artificial intelligence? What do primary health care stakeholders say? | 2022 | BMC medical informatics and decision making | Terry et al. | Y - RK | Y - AF | N - RK | N - JC | Diagnostic AI not explicitly investigated | Diagnostic AI not explicitly investigated | Diagnostic AI not explicitly investigated |
| Endoscopists’ acceptance on the implementation of artificial intelligence in gastrointestinal endoscopy: development and case analysis of a scale | 2022 | Frontiers in medicine | Tian et al. | Y - RH | Y - RK | N - RK | N - JS | Not primary qualitative research | Not primary qualitative research | Not primary qualitative research |
| Medical specialists’ perception about adoption of artificial intelligence in the healthcare sector | 2022 | Cardiometry | Thakkar and Bharathi | Y - RK | Y - AF | N - RK | N - AF | Not primary qualitative research | Not primary qualitative research | Not primary qualitative research |
| Attitude of colonoscopists towards artificial intelligence – a multinational study | 2022 | Gut | Thoufeeq et al. | N - RK | Y - AF | N - DJ | N - RK | Not primary qualitative research | Conference abstract | Conference abstract |
| Patients’ views of wearable devices and AI in healthcare: findings from the ComPaRe e-cohort | 2019 | NPJ digital medicine | Tran et al. | Y - RK | Y - AF | N - AF | N - RK | Diagnostic AI not explicitly investigated | Diagnostic AI not explicitly investigated | Diagnostic AI not explicitly investigated |
| Considerations for artificial intelligence real-world implementation in ophthalmology: providers’ and patients’ perspectives | 2021 | Asia-Pacific Journal of Ophthalmology | Tseng et al. | Maybe - RK | Y - RH | N - AF | N - RK | Systematic review | Not primary qualitative research | Systematic review |
| Priorities for artificial intelligence applications in primary care: a Canadian deliberative dialogue with patients, providers, and health system leaders | 2023 | J Am Board Fam Med | Upshaw et al. | Maybe – RK | Maybe – AF | N – AF | N - RK | Diagnostic AI not explicitly investigated | Diagnostic AI not explicitly investigated | Diagnostic AI not explicitly investigated |
| An exploration of expectations and perceptions of practicing physicians on the implementation of computerized clinical decision support systems using a Qsort approach | 2022 | BMC med inform decis mak | Van Biesen et al. | Y - RK | Y - AF | N - AF | N - RK | Diagnostic AI not explicitly investigated | Diagnostic AI not explicitly investigated | Diagnostic AI not explicitly investigated |
| Analyzing the determinants to accept a virtual assistant and use cases among cancer patients: a mixed methods study | 2022 | BMC Health Services Research | Van Bussel et al. | N - RK | Y - RH | N - RK | N - AF | Diagnostic AI not explicitly investigated | Diagnostic AI not explicitly investigated | Diagnostic AI not explicitly investigated |
| Attitude towards artificial intelligence of intensive care professionals and their perceived barriers for implementation: a survey study | 2022 | Intensive care medicine experimental Supplement | Van de Sande et al. | Y - RK | Y - AF | N - AF | N - RK | Conference abstract | Conference abstract | Conference abstract |
| Artificial intellilgence in (gastrointestinal) healthcare: patients’ and physicians’ perspectives | 2022 | Scientific reports | Van der Zander et al. | N - RK | Y - RH | N - DJ | N - RK | Not primary qualitative research | Not primary qualitative research | Not primary qualitative research |
| Rethinking the role of AI with physicians in oncology: revealing perspectives from clinical and research workflows | 2023 | Conference on human factors in computing systems – proceedings 2023 | Verma et al. | N – RK | Maybe – AF | N – RK | N - AF | Conference abstract | Conference abstract | Conference abstract |
| Barriers and facilitators to the adoption of artificial intelligence in radiation oncology: a New Zealand study | 2021 | Technical innovations and patient support in radiation oncology | Victor Mugabi | Y - RK | Y - AF | N - JS | N - RK | Not primary qualitative research | Not primary qualitative research | Not primary qualitative research |
| Physicians’ perspectives on AI in clinical decision support systems: interview study of the CURATE.AI personalised dose optimization platform | 2023 | JMIR human factors | Vijayakumar et al. | N – RK | Maybe – AF | N – AF | N - RK | Diagnostic AI not explicitly investigated | Diagnostic AI not explicitly investigated | Diagnostic AI not explicitly investigated |
| Community perspectives on AI/ML and health equity: AIM-AHEAD nationwide stakeholder listening sessions | 2023 | PLOS digital health | Vishwanatha et al. | Maybe – RK | Maybe – AF | N – AF | N - RK | Diagnostic AI not explicitly investigated | Diagnostic AI not explicitly investigated | Diagnostic AI not explicitly investigated |
| Gastroenterologist sentiment towards artificial intelligence (AI) in endoscopic practice: a nationwide survey | 2019 | American journal of gastroenterology | Wadhwa et al. | Y - AF | Y - RK | N - AF | N - RK | Conference abstract | Conference abstract | Conference abstract |
| Patients’ perceptions of integrating AI into healthcare: systems thinking approach | 2022 | IEEE | Wang et al. | Maybe - RK | Y - RH | N - RK | N - AF | Commentary/editorial | Not primary qualitative research | Not primary qualitative research |
| Ethical concerns with the use of intelligent assistive technology: findings from a qualitative study with professional stakeholders | 2019 | BMC Medical Ethics | Wangmo et al. | Y - RK | N - RH | N - RK | N - AF | Diagnostic AI not explicitly investigated | Diagnostic AI not explicitly investigated | Diagnostic AI not explicitly investigated |
| Perspective of information technology decision makers on factors influencing adoption and implementation of artificial intelligence technologies in 40 German Hospitals: descriptive analysis | 2022 | JMIR medical informatics | Weinert et al. | Y - AF | Y - RK | N - RK | N - JC | Not primary qualitative research | Not primary qualitative research | Not primary qualitative research |
| Perception and knowledge of artificial intelligence in healthcare, therapy and diagnostics: a population-representative survey | 2022 | medRxiv | Wittal et al. | Y - AF | Y - RK | N - AF | N - RK | Not peer-reviewed | Not peer-reviewed | Not peer-reviewed |
| Implementation of artificial intelligence in medicine: status analysis and development suggestions | 2020 | Artificial intelligence in medicine | Xiang et al. | Y - AF | Y - RK | N - RK | N - DJ | Not primary qualitative research | Not primary qualitative research | Not primary qualitative research |
| Do people favour artificial intelligence over physicians? A survey among the general population and their view on artificial intelligence in medicine | 2022 | Value in health: the journal of the international society for pharmacoeconomics and outcomes research | Yakar et al. | Y - RK | Y - AF | N - RK | N - AF | Not primary qualitative research | Not primary qualitative research | Not primary qualitative research |
| Attitudes of Chinese cancer patients toward the clinical use of artificial intelligence | 2019 | Patient preference and adherence | Yang et al. | Y - RK | Maybe - JC | N - RK | N - AF | Not primary qualitative research | Not primary qualitative research | Not primary qualitative research |
| Patients’ perceptions of artificial intelligence in diabetic eye screening | 2022 | Clinical and experimental ophthalmology | Yap et al. | Y - RK | Y - JS | N - DJ | N - RK | Conference abstract | Conference abstract | Conference abstract |
| Psychosocial factors affecting artificial intelligence adoption in health care in China: cross-sectional study | 2019 | Journal of medical internet research | Ye et al. | Y - RK | Maybe - JC | N - RK | N - JS | Not primary qualitative research | Not primary qualitative research | Not primary qualitative research |
| Healthcare professionals’ expectations for medical artificial intelligence and strategies for its clinical implementation: a qualitative study | 2023 | Healthcare informatics research | Yoo et al. | Y - RK | Y - AF | N - JC | N - RK | Diagnostic AI not explicitly investigated | Diagnostic AI not explicitly investigated | Diagnostic AI not explicitly investigated |
| Clinician and computer: a study on doctors’ perceptions of artificial intelligence in skeletal radiology | 2023 | BMC medical education | York et al. | Y - RK | Y - RH | N - RK | N - JS | Not primary qualitative research | Not primary qualitative research | Not primary qualitative research |
| Barriers and facilitators to implementing a nursing clinical decision support system in a tertiary hospital setting: a qualitative study using the FITT frameowrk | 2022 | International journal of medical informatics | Zhai et al. | N - RK | Y - RH | N - RK | N - SD | Diagnostic AI not explicitly investigated | Diagnostic AI not explicitly investigated | Diagnostic AI not explicitly investigated |
| The adoption of AI in mental health care-perspectives from mental health professionals: qualitative descriptive study | 2023 | JMIR formative research | Zhang et al. | Maybe – AF | Maybe – RK | N – RK | N - AF | Diagnostic AI not explicitly investigated | Diagnostic AI not explicitly investigated | Diagnostic AI not explicitly investigated |
| Lay individuals’ perceptions of artificial intelligence (AI)-empowered healthcare systems | 2020 | Proceedings of the association for information science and technology | Zhang et al. | Y - AF | Y - RK | N - RK | N - AF | Not primary qualitative research | Not primary qualitative research | Not primary qualitative research |
| Exploring patients’ AI adoption intention in the context of healthcare | 2021 | Digital Health and Medical Analytics | Zhu and Sun | Maybe - RK | Y - AF | N - DJ | N - RK | Not primary qualitative research | Conference abstract | Conference abstract |
| Attitude and perception of radiology staff towards artificial intelligence | 2022 | Clinical radiology | Zhang et al. | Y - RK | Y - AF | N - RK | N - JC | Not primary qualitative research | Conference abstract | Conference abstract |
| Patients’ perceptions of using artificial intelligence (AI)-based technology to comprehend radiology imaging data | 2021 | Health informatics journal | Zhang et al. | Y – RK | Y - AF | N - RK | Y - RH | Diagnostic AI not explicitly investigated |  | Diagnostic AI not explicitly investigated |
| Attitudes of medical workers in China towards artificial intelligence in ophthalmology: a comparative survey | 2021 | BMC health services research | Zheng et al. | Y - AF | Maybe - RK | N - RK | N - AF | Not primary qualitative research | Not primary qualitative research | Not primary qualitative research |
| Psychiatrists’ views on robot-assisted diagnostics of peripartum depression | 2021 | Social robotics | Zhong et al. | Y - RH | Y - AF | N - SD | N - RK | Conference abstract | Conference abstract | Conference abstract |
